# Effectiveness of strip footing with geogrid reinforcement for different types of soils in Mosul, Iraq

**DOI:** 10.1371/journal.pone.0243293

**Published:** 2020-12-17

**Authors:** Noor Ibrahim Hasan, Aizat Mohd Taib, Nur Shazwani Muhammad, Muhamad Razuhanafi Mat Yazid, Azrul A. Mutalib, Dayang Zulaika Abang Hasbollah

**Affiliations:** 1 Faculty of Engineering & Built Environment, Universiti Kebangsaan Malaysia, Bangi UKM, Selangor, Malaysia; 2 School of Civil Engineering, Faculty of Engineering, Universiti Teknologi Malaysia, Skudai, Johor, Malaysia; China University of Mining and Technology, CHINA

## Abstract

The main cause of problematic soil failure under a certain load is due to low bearing capacity and excessive settlement. With a growing interest in employing shallow foundation to support heavy structures, it is important to study the soil improvement techniques. The technique of using geosynthetic reinforcement is commonly applied over the last few decades. This paper aims to determine the effect of using geogrid Tensar BX1500 on the bearing capacity and settlement of strip footing for different types of soils, namely Al-Hamedat, Ba’shiqah, and Al-Rashidia in Mosul, Iraq. The analysis of reinforced and unreinforced soil foundations was conducted numerically and analytically. A series of conditions were tested by varying the number (*N*) and the width (*b*) of the geogrid layers. The results showed that the geogrid could improve the footing’s bearing capacity and reduce settlement. The soil of the Al-Rashidia site was sandy and indicated better improvement than the other two sites’ soils (clayey soils). The optimum geogrid width (*b*) was five times the footing width (*B*), while no optimum geogrid number (*N*) was obtained. Finally, the numerical results of the ultimate bearing capacity were compared with the analytical results, and the comparison showed good agreement between both the analyses and the optimum range published in the literature. The significant findings reveal that the geogrid reinforcement may induce improvement to the soil foundation, however, not directly subject to the width and number of the geogrid alone. The varying soil properties and footing size also contribute to both BCR and SRR values supported by the improvement factor calculations. Hence, the output complemented the benefit of applying reinforced soil foundations effectively.

## Introduction

Ground improvement methods by using geosynthetics have been developed extensively over the last few decades, particularly in the applications of pavement and foundation engineering. Although many experimental studies were undertaken to determine the effect of geosynthetic reinforcement, the analysis differs in regards to the properties of the geotextile such as shapes and sizes, spacing and thickness [1–13]. On top of that, the studies also analyse the influence of different types of soils and footing designs. In regards to the soil behaviour with sandy soil classification, numerous analytical studies have contributed to the knowledge of soil-structure interaction conducted by several researchers towards the bearing capacity of geogrid-reinforced soil foundations [13–17]. Additionally, innumerable numerical models which time and cost-saving have been carried out to investigate the bearing capacity and the settlement of reinforced soil [9, 18–29]. The concept of reinforced soil as construction material which is based on the existence of soil-reinforcement interactions due to tensile strength, frictional, and adhesion properties of the reinforcement was first introduced by the French architect and engineer Henri Vidal in the 1960s [29]. Since then, this technique has been widely used in geotechnical engineering practice. Geosynthetics, which is used in reinforced soils come in many types which include the geogrids, geotextiles, geomembranes, geosynthetic clay liners, geonets, and geocells [30]. Geogrid is one of the planer geosynthetic products normally made of polymers; nowadays different varieties of geogrids are made of polypropylene or high-density polypropylene (HDPP) and that promotes the influence of different geotextile materials in functioning effectively.

**Fig 1:**
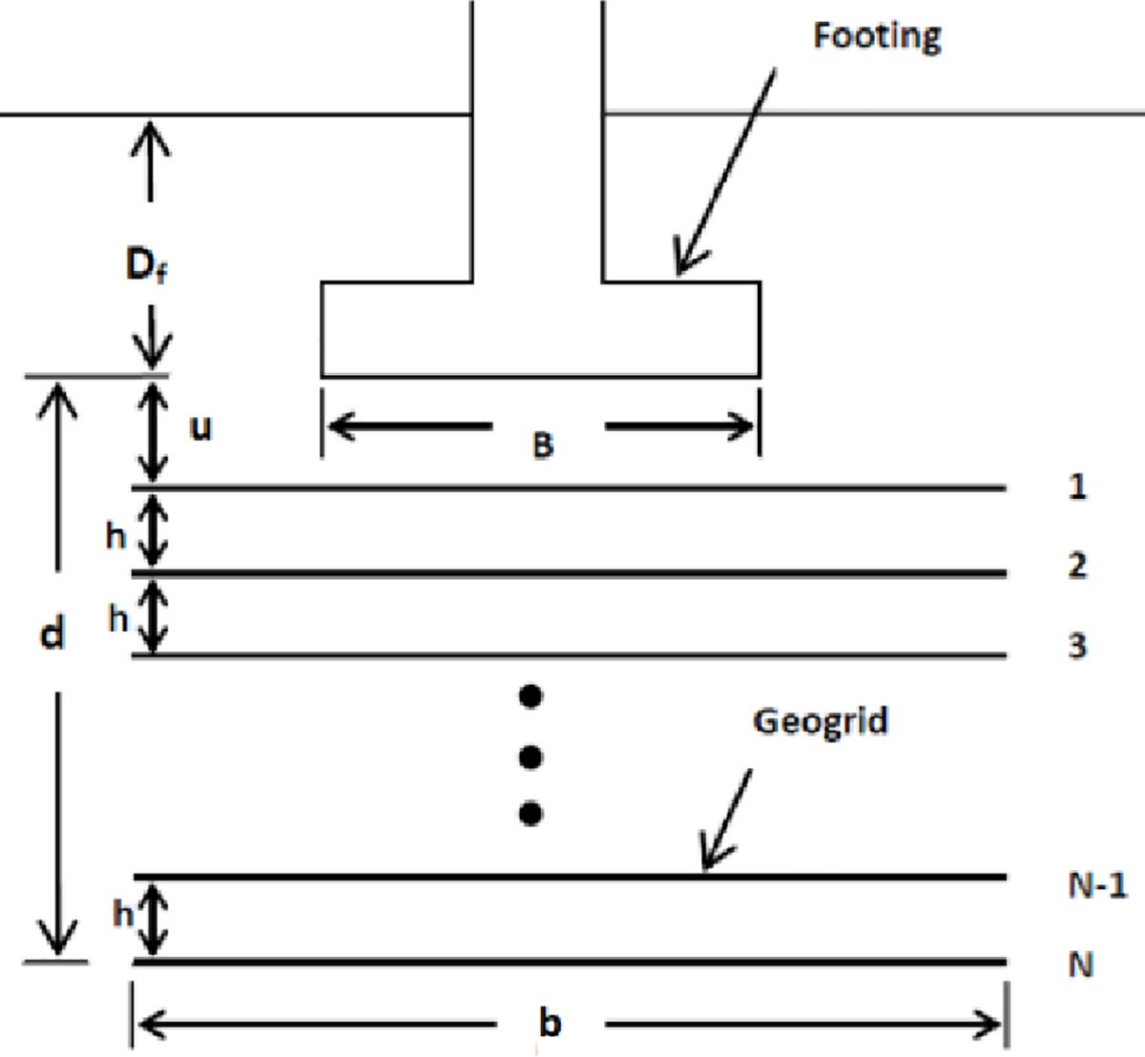
Geogrid reinforced soil foundation [32].

A foundation with the reinforced-soil system is called reinforced soil foundation (RSF). Fig 1 illustrates a typical geosynthetic reinforced soil foundation and the description of various geometric parameters. The parameters of the geogrid reinforcement involve top layer spacing (*u*), vertical spacing (*s* or *h*), number of reinforcement layers (*N*), total depth of reinforcement (*d*), and width of reinforcement (*b*). As indicated in the literature, the optimum value for (*u*/*B*) and (*h*/*B*) parameters is 0.33 (where B is the footing width). Many studies have opted different dimension for the footing and geogrid yet all findings are pointing out to different behaviour depending on the soil classification. It can be understood that dissimilar geographic locations have different soil types and conditions, hence, a proper design of geogrid utilised is important in enhancing the soil foundations. Moreover, reinforced soil foundations could be an economical alternative to conventional shallow foundations with large footing dimensions which in turn increase foundation settlement due to an increase in the depth of influence zone below the foundation, or replacement of weak soil layers with competent materials [31].

During the past thirty years, many experimental, numerical, and analytical studies have been performed to investigate the behaviour of RSF for different soil types. All studies indicated that the use of reinforcements can significantly increase the bearing capacity and reduce the settlement of soil foundations [33]. Chen & Abu-Farsakh *et al*. [34] work has utilised two concepts to evaluate the benefits of reinforced soil foundation, for example, the bearing capacity ratio (BCR) and settlement reduction ratio (SRR). BCR is defined as the ratio of the bearing capacity of the reinforced soil foundation to that of unreinforced soil foundation whereas SRR is defined as the ratio of the reduction in footing settlement based on reinforcement to the settlement of unreinforced soil foundation at a constant surface pressure [35]. BCR is given as:

$$BCR=\frac{{\left(q_{ult}\right)}_{r}}{{\left(q_{ult}\right)}_{u}}$$ (1)

Where:

(*q*_*ult*_)_*r*_ is the ultimate bearing capacity of reinforced soil foundation.

(*q*_*ult*_)_*u*_ is the ultimate bearing capacity of unreinforced soil foundation.

And SRR is given as:

$$SRR=\frac{s_{0}-s_{R}}{s_{0}}$$ (2)

Where:

*s*_*R*_ is the settlement of reinforced soil foundation.

*s*_0_ is the settlement of unreinforced soil foundation.

Many of these research efforts were aimed at investigating the parameters and variables that would contribute to the BCR and SRR values. Other research also focused on improving the settlement of footing, other geotechnical structures and calculation methods such as Abbas *et al*. [36], Rosyidi *et al*. [37], Khajehzadeh *et al*. [38], Joh *et al*. [39], Chik *et al*. [40], Li *et al*. [41], Azrief *et al*. [42] and Zhanfang *et al*. [43] work. Guido *et al*. [1] conducted an experimental study on geotextile-reinforced earth slabs. Their model tests were conducted using square footing on sand. They showed that the BCR decreased with an increase in *u/B*; the improvement in the bearing capacity was negligible when the number of reinforcement layers was increased beyond three, which corresponded to an influence depth of *1*.*0B* for the *u/B*, *h/B*, and *b/B* ratios of 0.5, 0.25, and 3. Negligible improvement on the BCR was observed while increasing the length ratio (*b/B*) of the reinforcement beyond three with two reinforcement layers and *u/B* and *h/B* ratios of 0.25 and 0.25, respectively. Furthermore, Lee *et al*. [44] conducted a laboratory model test by using a rigid strip footing supported on the dense sand overlying the soft clay with a layer of geotextile reinforcement at the interface. They found that a reinforcement layer at the sand–clay interface resulted in an additional increase in the bearing capacity and a decrease in the settlement of the footing; the effective width of the reinforcement that resulted in the optimum performance of the footing was found to be approximately five to six times the width of the footing.

Additionally, a finite element analysis study by Kurian *et al*. [45] on a strip footing supported by reinforced sand using the Duncan–Chang soil model showed a clear reduction of settlement in the reinforced sand at higher loads than in the case of the unreinforced sand. The numerical results also indicated that a small increase in settlement occurred in the reinforced sand in the initial stage of the loading process. A possible explanation of this phenomenon given by Kurian *et al*. [45] was that the normal load was too small to mobilise sufficient friction between the soil and the reinforcement. The relative movement between the soil and the reinforcement increased with an increase in the load and decreased with an increase in the reinforcement depth. The maximum shear stress at the soil–reinforcement interface occurred at a relative distance (*x/B*) of approximately 0.5 from the centre of the footing, and the tension developed in the reinforcement was maximum at the centre and gradually decreased toward the end of the reinforcement. On the other hand, Maharaj [19] performed a numerical analysis on a strip footing supported by reinforced clay by using the Drucker–Prager soil model. He concluded that in the case of a single layer of reinforcement, the optimum top layer spacing ratio (*u/B*) was found to be around 0.125 in reinforced clay. He also found that the effective length ratio (*b/B*) of reinforcement was around 2.0, the influence depth depended on the stiffness of reinforcement, and the increase in the geosynthetic stiffness reduced the settlement of the footing.

Although many studies showed many interesting features of the soil-geosynthetics interaction mechanism, the methods used for the design of geosynthetic-reinforced soil systems still vary and most of the time puzzling to engineers. The design of reinforced soil system using limit equilibrium methods was mostly used and considered to be very conservative [46–48]. Recently, the adoption of finite element method for modelling and analysing the reinforced soil system has provided appropriate design specifications, low cost and speed, utilising different soil-reinforcement system and boundary conditions [49]. However, the need for a numerical and analytical study that consider the main factors of the interaction mechanism for reinforced soil foundation remains essential. In this paper, the analysis of bearing capacity and settlement of geogrid reinforced and unreinforced soil foundation of the three sites (i.e. Al-Hamedat, Al-Rashidia, and Ba’shiqa) in Mosul, Iraq is conducted numerically via the finite element program Plaxis and compared to the analytical bearing capacity computed theoretically by using a method derived by Chen and Abu-Farsakh [17]. The derived and analytical methods are based on limit equilibrium analysis and only compute the ultimate bearing capacity in regards to a given settlement. Since no settlement can be obtained from these methods, therefore, the settlements obtained from the numerical analysis were utilised in the theoretical method.

## Mechanism of geogrid reinforcement

In many cases of construction, shallow foundations are built on top of the existing weak soil, and this results in a low bearing capacity and excessive settlement problems. The drawbacks can cause structural damage, reduction in durability, and deterioration in the performance level [50]. Under these conditions, soil improvement techniques have been used for a long time to overcome the problem in these types of soils. Several researchers have developed various soil improvement techniques to improve soil strength by using different stabilising methods. Several types of soil improvement methods, including grouting, vertical drains, soil replacement, piling, and geosynthetic reinforcement, have been developed to solve the aforementioned soil problems [51–54]. The polymeric nature of the geosynthetic material makes geosynthetic products durable under different ground and environmental conditions. Common applications of geosynthetics in the field of geotechnical engineering include improving strength and stiffness of the subsurface soil underlined at shallow foundations and pavements, providing stability to earth retaining structures and slopes, ensuring dam safety, as discussed in Han *et al*. [55] and Wang *et al*. [56] work. Geogrid is used for improving the mechanical performance of subsurface soil under external loadings. Thus, it is widely applied as reinforcement layers in mechanically stabilised earth (MSE) and geosynthetic reinforced soil (GRS) walls, as a measure of slope stabilisation and as reinforcement in subsurface soil below pavements and footings. The high tensile capacity of geogrids allows the reinforcement layers to take over a significant part of tensile stresses generated within a soil mass because of the action of external loading. Therefore, geogrids act as reinforcing elements and enhance the load–deformation behaviour of the reinforced soil mass.

In the highlights of some experimental studies, Binquet and Lee [14] evaluated bearing capacity of the soil reinforced with metal strips; the test results indicated that the bearing capacity could be improved by a factor ranging from 2 to 4 by reinforcing the soil. Their test results also suggested that the reinforcement placed below the influence depth, which was approximately *2B*, had a negligible effect on the increase in the bearing capacity, and placing the first layer at (*u/B* = 0.3) below the base of the footing resulted in the maximum improvement. Akinmusuru and Akinbolade [57] investigated the influences of using rope fibres as reinforcing elements on sandy soil; their results showed that the ultimate bearing capacity could be improved by a factor of up to three times that of the unreinforced soil; the optimum top layer spacing (*u*) was determined to be *0*.*5B*, and they showed that the improvement in the bearing capacity was negligible when the number of reinforcement layers was increased beyond three, which corresponded to an influence depth of *1*.*75B*. Sakti and Das [2] conducted an experimental study on the geotextile-reinforced clayey soil foundation. Their test results demonstrated that most of the benefits of the geotextile reinforcement were obtained at a top layer spacing ratio (*u/B*) of 0.35 to 0.4. For *u/B* of 0.33 and *h/B* of 0.33, the BCR increased from 1.1 to 1.5 when the number of layers increased from 1 to 3 and remained practically constant thereafter. The influence depth of placing the geotextile was then determined to be 1.0*B*. The most effective length of the geotextile was equal to four times the width of the strip footing

Zhou and Wen [58] conducted an experimental study to investigate the effect of using a single layer of geocell-reinforced sand cushion on soft soil. The results indicated that there was a substantial reduction in the settlement of the underlying soft soil, and the subgrade reaction coefficient *K30* was improved by 3000%; the deformation was reduced by 44%. Moreover, Raftari *et al*. [24] conducted a numerical analysis on the strip footing supported by the reinforced slope by using the Mohr–Coulomb soil model. The test results showed that the foundation settlement on the unreinforced slope is more severe than on the reinforced slope. As the settlement in the reinforced situation with three layers of reinforcement decreased by approximately 50%. They reported that to obtain the least settlement, the optimum vertical spacing between the geogrids (*h*) should be equivalent to the width of the foundation (*B*). Khing *et al*. [5] conducted a series of model tests on strip footings supported by the geogrid reinforced sand. The test results indicated that placing the geogrid at a depth ratio (*d/B*) greater than 2.25 resulted in no improvement on the bearing capacity of the strip footing. To achieve maximum benefit, the minimum length ratio (*b/B*) of the geogrid should be equal to 6. The BCR calculated at a limited settlement ratio (*s/B*) of 0.25, 0.5, and 0.75 was approximately 67%–70% of the ultimate BCR.

Adams and Collin [11] performed several series of large-scale field tests. The tests were conducted in a concrete box with four different sizes of square footings. Poorly graded fine concrete mortar sand was selected for the tests. The test results indicated that three layers of geogrid reinforcement could significantly increase the bearing capacity and that the ultimate bearing capacity ratio (BCR) could be increased to more than 2.6 for three layers of reinforcement. However, the amount of settlement required for this improvement was approximately 20 mm (*s/B* = 5%) and might be unacceptable on some foundation application. The results also showed that the beneficial effects of reinforcement at a low settlement ratio (*s/B*) can be achieved maximally when the top layer spacing is less than 0.25*B*. Alternatively, Arab *et al*. [27] conducted a numerical analysis on the strip footing supported by sandy soil by using a hardening soil model. They reported that for the geometrical parameters *u*/*B* = *h*/*B* = 0.5, and *b*/*B* = 4, the effect of the increasing number of geogrid layers (*N*) on the bearing capacity of the geogrid reinforced soils increased the bearing capacity and slightly increased the overall stiffness of the reinforced sand. The increment of the geogrid stiffness resulted in a BCR increase as well. Although the studies in geogrid-reinforced soil foundation have been widely undertaken yet the soil behaviour is not entirely captured particularly involving an optimised application of the geogrid. The numerical modelling in this study promotes deeper understanding of soil foundation via the specification of reinforcement in the soil models.

## Numerical modelling

The numerical modelling of reinforced and unreinforced soil foundation behaviour was conducted by using the Plaxis software. Plaxis is a finite element program specially developed for the analysis of deformation and stability in geotechnical engineering problems [59]. In this study, the test process includes the full modelling of soil, geogrid reinforcement, installation of footing, and imposing loading as shown similarly in Fig 1. Real scenarios can be modelled with a plane strain model, which is used in the current problem. The plane strain model is suitable to implement with a relatively uniform cross-section, loading scheme and a great extent of the model in the direction perpendicular to the model plane where, normal stresses are fully considered but the displacements and strains are assumed to be zero.

### Model analysis

Different soil constitutive models are available in Plaxis. With the use of finite element modelling, the elastic-perfectly plastic Mohr–Coulomb soil model was considered in this study. Mohr-Coulomb constitutive model is widely used in the most of geotechnical engineering problems as researchers have shown that stress combinations leading to failure in soil samples in triaxial tests match the failure contour of Mohr-Coulomb criterion (hexagonal shape) Goldscheider [60]. When using the Mohr-Coulomb constitutive model, five parameters are required as input [61]. These five parameters can be retrieved by analyzing basic soil tests, and they constitute of two stiffness parameters: effective Young modulus (*E*′) and effective Poisson ratio (*v*′) and three strength parameters: effective cohesion (*c*′), effective friction angle (*φ*′), and dilation angle (*ψ*). In 2D space, the failure envelope symbolizes the straight or slightly curved line touching the Mohr circle or stress points. At stress ranges within the yield locus, the soil material is elastic in its behaviour. As a critical combination of shear stress and effective normal stress develops, the stress point will coincide with the failure envelope and perfectly plastic material behaviour is assumed, with continuous shearing at constant stress. Once a perfectly plastic state has been reached, the material can never return to a fully elastic behaviour without any irrecoverable deformations. The strip foundation is modelled as a rigid plate and is considered to be very stiff and rough in the analyses.

**Fig 2:**
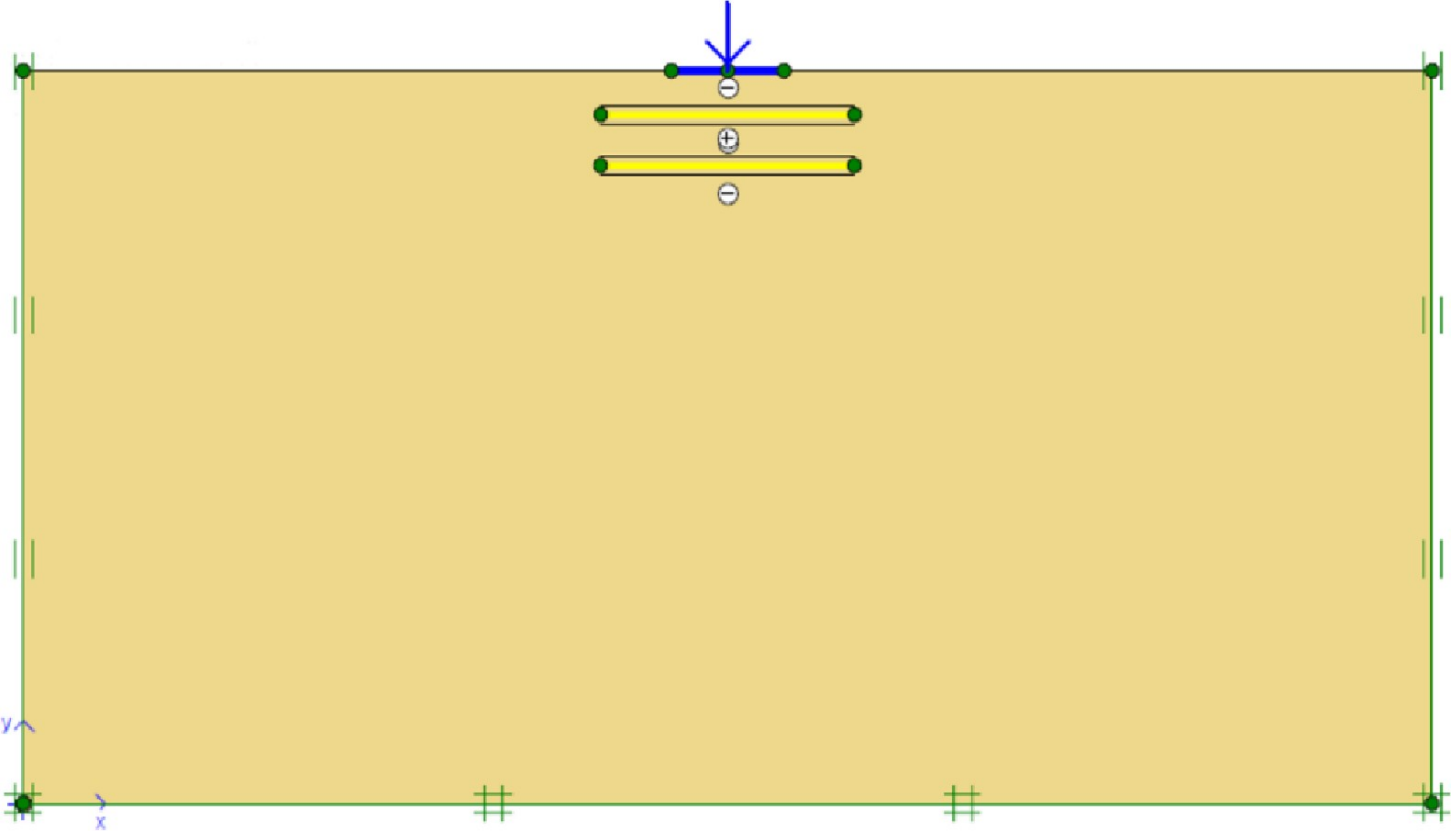
Interfaces, geogrids, footing, point load, and the standard fixities available in Plaxis.

The details of geogrid reinforced soils considered in the model tests are shown in Table 1. In Plaxis, geogrid reinforcements are represented by the use of special tension elements (five-node geogrid elements). Geogrids have only normal stiffness and no bending stiffness which can only sustain the tensile forces. The only material property of a geogrid is an elastic axial stiffness *EA*. To model the interaction of the geogrid elements with the surrounding soil, it is often convenient to combine these geogrid elements with interfaces. The assigned soil–geogrid interfaces are shown in Fig 2. Each interface has assigned to it a virtual thickness which is an imaginary dimension used to define the material properties of the interface. An elastic-perfectly plastic model is used to describe the behaviour of interfaces for the modelling of soil-geogrid interaction. The coulomb criterion is used to distinguish between elastic behaviour, where small displacements can occur within the interface and plastic interface behaviour when permanent slip occurs. The interface parameters are calculated from the surrounding soil parameters adopting the interaction coefficient *R*_*inter*_, defined as the ratio of the shear strength of the interface to the shear strength of the soil [59]. In this study 15-node soil elements are used, and the interface strength is set to manual. For real soil-structure interaction, the interface is weaker and more flexible than the associated soil, which means that the value of *R*_*inter*_ should be less than 1. Therefore, *R*_*inter*_ is assumed to be 0.9 in the present study.

**Table 1:** Details of model test program.

Test series	Constant parameters	Variable Parameters
A	*u/B* = 0.33, *N* = 1	*b/B* = 1, 2, 3, 4, 5, 6
B	*u/B* = *h/B* = 0.33, *N* = 2	*b/B* = 1, 2, 3, 4, 5, 6
C	*u/B* = *h/B* = 0.33, *N* = 3	*b/B* = 1, 2, 3, 4, 5, 6
D	*u/B* = *h/B* = 0.33, *N* = 4	*b/B* = 1, 2, 3, 4, 5, 6
E	*u/B* = *h/B* = 0.33, *N* = 5	*b/B* = 1, 2, 3, 4, 5, 6

**Fig 3:**
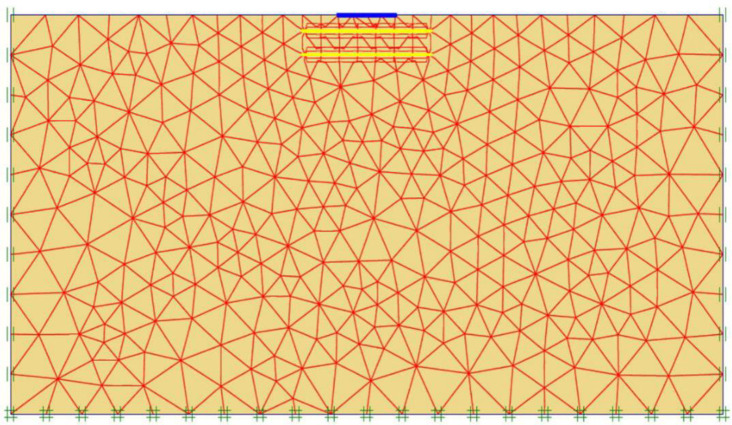
Finite element mesh of the reinforced soil mode.

**Fig 4:**
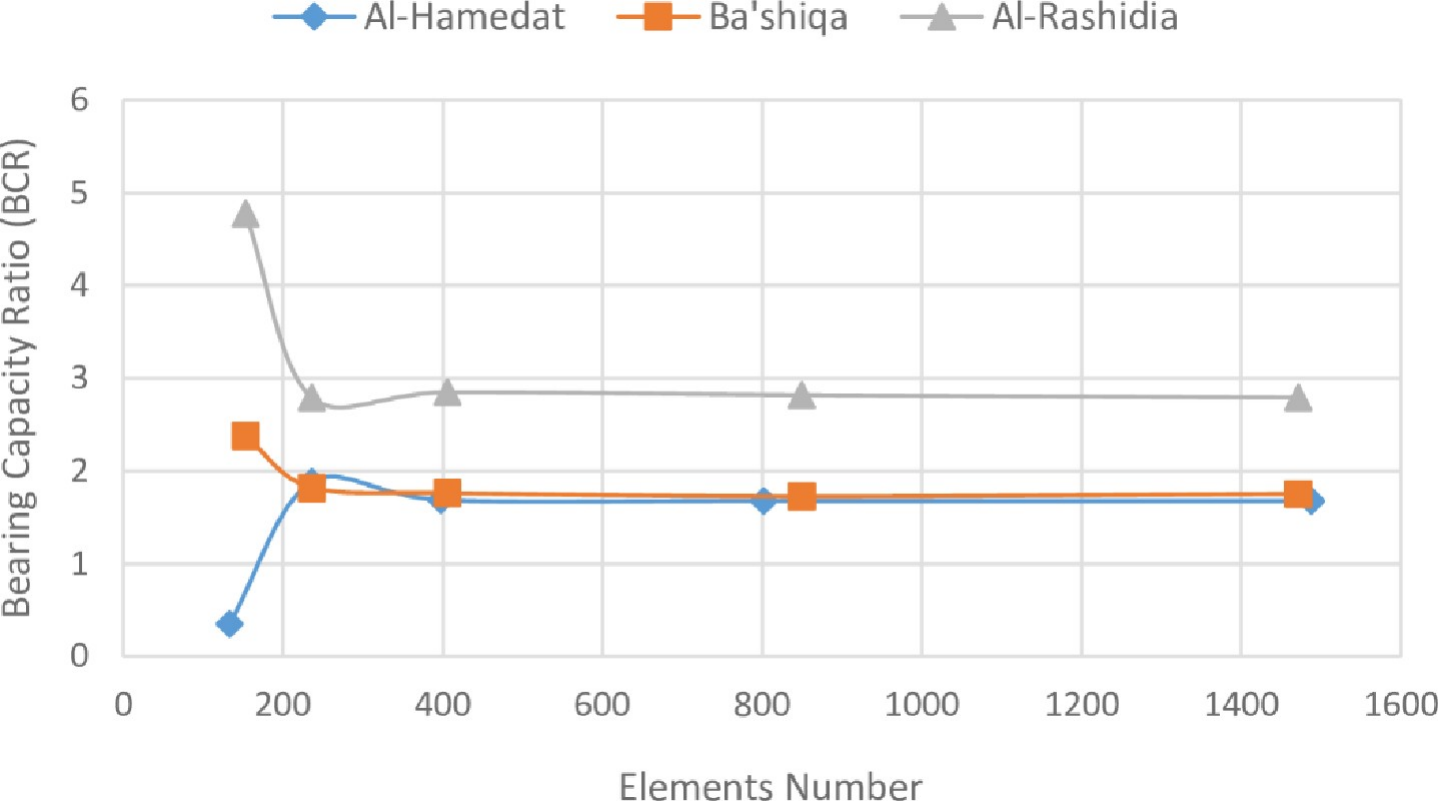
Variation of the bearing capacity ratio with the mesh density (mesh coarseness).

After the geometry model is fully defined and material properties are assigned to soil layers and structural objects, the mesh is applied for finite element (FE) calculations. Plaxis incorporates a procedure of fully automatic mesh generation, in which the geometry is discretised into elements of the basic element type, and compatible structural elements as shown in Fig 3. The basic type of element in a mesh used in the present study is the triangular element with average size of 0.5 to 2 m which provides an accurate calculation of stress and failure loads. Five different mesh densities are available in Plaxis, ranging from very coarse to very fine. Preliminary computations were conducted using the five available levels of global mesh coarseness in order to obtain the most suitable mesh density and to minimise the effect of the mesh dependency on the finite element modelling. In the analysis, the number of triangular elements and stress points in the model for each site was changed depending on the mesh density and the reinforcement arrangements. Table 2 shows the elements and stress points numbers variation with mesh density of the three sites models for the case of five geogrid layers. As seen in Fig 4, the mesh size has minimum effect on the results after about 240 elements for Ba’shiqa site and 400 elements for both Al-Hamedat and Al-Rashidia sites. For Ba’shiqa this corresponds to the coarse mesh with refinement around the geogrid elements and model foundation where large stress concentrations are expected and medium mesh with refinement for both Al-Hamedat and Al-Rashidia.

**Table 2:** Elements and stress point numbers variation with the mesh density.

Mesh Coarseness	Al-Hamedat		Ba'shiqa		Al-Rashidia	
	Element	Stress Points	Element	Stress Points	Element	Stress Points
Very Course	133	1596	153	1836	153	1836
Course	236	2832	236	2832	236	2832
Medium	398	4776	406	4872	406	4872
Fine	802	9624	850	10200	850	10200
Very Fine	1488	17856	1472	17664	1472	17664

**Fig 5:**
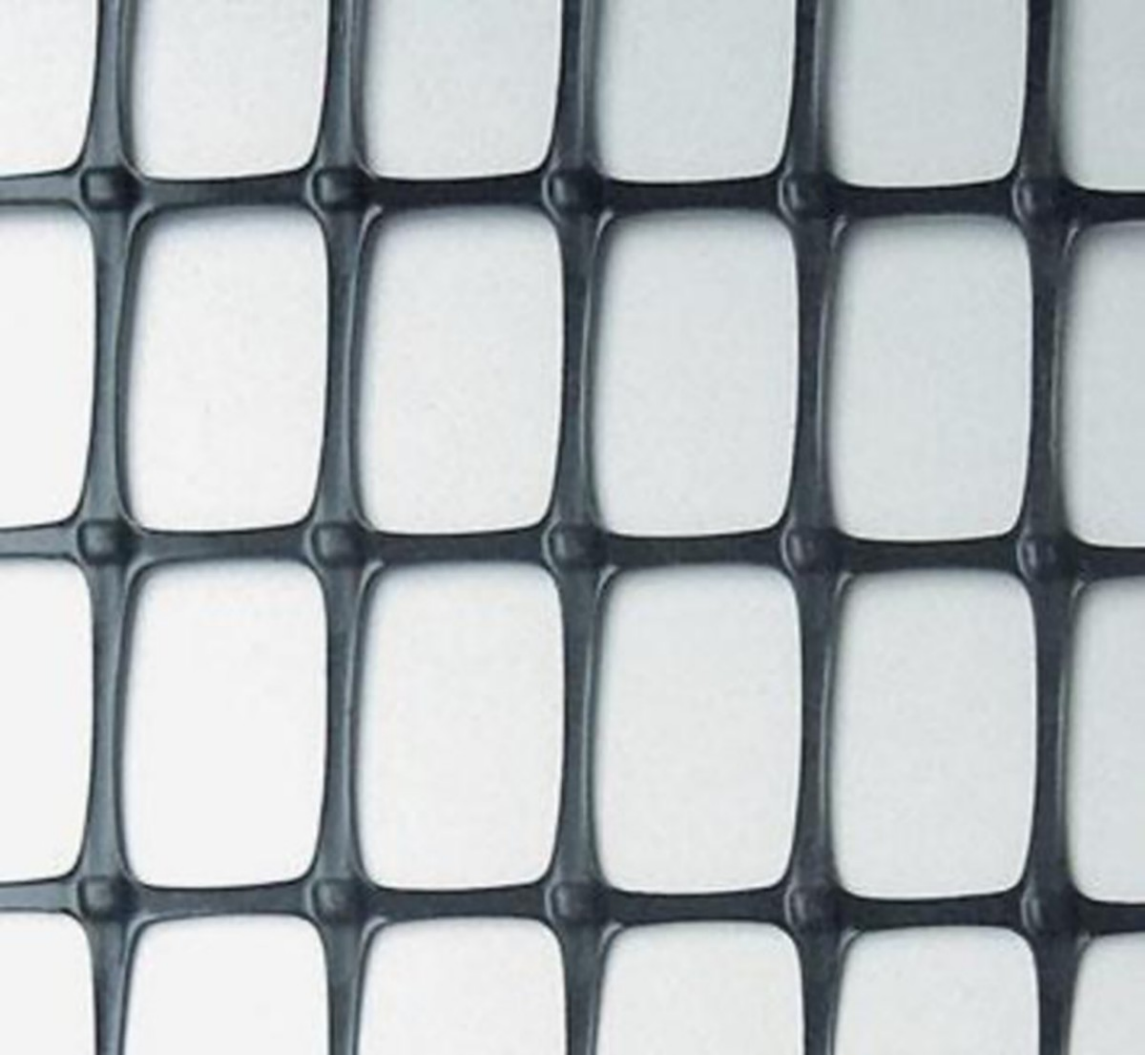
Polymeric extruded biaxial geogrid type BX1500 [62].

The modelled boundary conditions were assumed such that the vertical boundaries were free vertically and constrained horizontally, while the bottom horizontal boundary was fully fixed, as shown in Fig 5. The considered vertical boundaries of the mesh were 10 m away from the center of the foundation on each side while the bottom horizontal boundary was 20 m below the foundation base such that these boundaries do not influence the stresses and deformations generated within the soil mass. Point load was utilised in the study. The structure was modelled with an increasing magnitude of loading until the soil reached a failure to examine the settlement influenced by the imposed load. Once the geometry model has been created and the finite element mesh being generated, the initial stress state must be specified. The initial conditions consist of two different modes: One mode for the generation of initial water pressure and the other mode for the specification of the initial geometry configuration and generation of the initial effective stress field. As the soil layers for Al-Hamedat and Ba’shiqa are dry and the water table at the Al-Rashidia site is deep enough to not affect the foundation behaviour, the groundwater condition was assumed to be negligible. The initial stresses in the soil are generated using Jaky’s formula, expressed by Eq 3 (in Plaxis software, the procedure to generate initial soil stresses is often known as the *K*_0_ procedure).

$$K_{0}=1-sin\;(\varphi )$$ (3)

where *K*_0_ is the lateral earth pressure coefficient and *φ* is the internal friction angle of the soil.

Plaxis allows for different types of finite element calculations such as plastic calculation, consolidation analysis, Phi-c reduction analysis, and dynamic calculation. For the current study, a plastic calculation has been selected. A plastic calculation should be selected to carry out an elastic-plastic deformation analysis. This type of calculation is appropriate in most practical geotechnical applications. In engineering practice, a project is divided into project phases. Similarly, a calculation process in Plaxis is also divided into calculation phases. In this study, two calculation phases are considered. The first one is the initial phase, which represents the initial situation of the problem. The second phase includes the inclusion of geogrid reinforcement and the external line load application.

In a finite element calculation, the analysis becomes non-linear when the plastic calculation is involved which means each calculation phase need to be solved in calculation steps (load steps). The step size and the solution algorithm is important to the non-linear solution. If the calculation step of a suitable size then the number of iterations required for equilibrium will be small about 5–10, while if the step is large then the number of iterations required will be excessive and the solution may diverge. The iterative parameters in the software: Desired minimum and maximum are primarily meant to determine when the calculation should take larger steps or smaller steps. If the calculation can solve a load step (hence converge) in fewer iterations than the desired minimum which by default is 4, it starts using a load step that is twice as big. If, however, the calculation needs more iterations than the desired maximum which by default is 10 to converge, the calculation will decide to choose a calculation step only half the size. For a Plastic analysis, there is no influence on the results when changing the desired minimum or desired maximum. As long as the calculation converges every step it is unimportant if the calculation uses a lot of small steps with few iterations or a limited number of larger steps with more iterations per step.

Several procedures are available for the solution of non-linear plasticity problems. All procedures are based on an automatic step size selection depending on the applied algorithm. Load advancement ultimate level is one of these procedures which is used in the current analysis. The automatic step size procedure is used primarily for calculation phases where a particular ultimate load level has to be reached. The procedure terminates the calculation when the specified load level is reached, or when soil failure is detected. The number of additional steps is set to 1000 to make the calculation process continue to the end before the number of additional steps is reached. In this procedure, the iterative parameters are set to standard and showed a good performance in the calculation convergence. In the standard settings the tolerated error which is the drift from the exact solution was set to 0.03, the over-relaxation factor which is responsible to reduce the number of iterations needed for convergence was set to 1.2, the maximum iterations were set to 50, the desired minimum and maximum iteration was set to 4 and 10 respectively, and finally, the arc-length control was activated which is important for the calculation to converge and to accurately determine the failure load else, the calculation will keep iterating and the failure load will be overestimated. The staged construction was selected as a loading input option where the value and configuration of the load, and the state of failure that need to be reached can be defined. Since staged construction is performed by using the load advancement ultimate level procedure, it is controlled by a total multiplier (∑Mstage). This multiplier generally starts at zero and reach the ultimate level of 1.0 at the end of the calculation phase. The time interval of the calculation phase considered to be zero as the model analysis is a plastic analysis, and does not include the consolidation or the use of soft soil creep model.

### Material properties

The soils were collected from three different sites of Mosul, Iraq, which were Al-Hamedat, Ba’shiqa, and Al- Rashidia. Mosul is located at the northern part of Iraq. The area is characterized by extensive plains and anticlines. Near the Tigris river, there are three levels of accumulated terraces of alluvial soils. Most of the soil in the area is of moderate expansive type. Flat areas between the anticlines are covered by sheet run-off sediments which include clay, sand, silt, and sometimes coated by scattered gravels. Table 3 shows the mechanical and physical soil properties, and S1 Table shows the Atterberg limits and grain size of each site involved. In this study, a concrete strip footing with width *B* = 600 mm was used. The properties of the footing are shown in Table 4. The biaxial geogrids (Tensar BX1500) shown in Fig 5 were used to reinforce the soil at all three sites. The various properties of geogrid reinforcement used in the finite element modelling of this study are shown in Table 5.

**Table 3:** Soil properties of the three sites from laboratory tests.

Location	Shear strength parameters		Physical soil properties				
	Angle of friction, *φ*°	Cohesion, *C* (kpa)	Saturated unit weight, *γ*_*sat*_ (kN/m^3^)	Unsaturated unit weight, *γ*_*unsat*_ (kN/m^3^)	Modulus of elasticity, *E* (kN/*m*^2^)	Poisson’s ratio *v*	Dilation angle *ψ*°
Al-Hamedat	20	40	20	17	25000	0.35	0
Ba’shiqah	25	15	17.5	15	32500	0.35	0
Al-Rashidia	28	0	20	16	32500	0.35	0

**Table 4:** Concrete footing properties used in numerical analysis.

Parameter	Unit	Value
Material Model	-	Linear Elastic
Unsaturated unit weight, *γ*_*unsat*_	kN/*m*^2^	24
Young’s Modulus (E)	kN/*m*^2^	21.5x10^6^
Poisson’s Ratio	-	0.3

**Table 5:** Physical and mechanical properties of geogrid used in this study.

Description	Unit	Geogrid BX1500
Polymer material	-	polypropylene
Aperture dimensions	mm (in)	30.5 (1.2)
Minimum Rib Thickness	mm (in)	1.78 (0.07)
Tensile Strength at 2% Strain	kN/m (Ib/ft)	10.0 (690)
Tensile Strength at 5% Strain	kN/m (Ib/ft)	20.0 (1,370)
Ultimate Tensile Strength	kN/m (Ib/ft)	30.0 (2,050)
Junction Efficiency	%	93
Flexural Stiffness	mg-cm	2000000
Aperture Stability	m-N/deg	0.75

### Ultimate bearing capacity of unreinforced soil foundation

Meyerhof [63] suggested a method to estimate the ultimate bearing capacity of strip footing including the depth factor (*D*_*f*_) as:

$$q_{u}=cN_{c}\;F_{cd}+qN_{q}\;F_{qd}+0.5\;\gamma \;B\;N_{\gamma }\;F_{\gamma d}$$ (4)

The bearing capacity factors can be given by the following relationships [63]:

$$N_{q}={tan}^{2}(45+\frac{\varphi }{2})e^{\pi \;tan\varphi }$$ (5)

$$N_{c}=cot\varphi (N_{q}-1)$$ (6)

$$N_{\gamma }=(N_{q}-1)tan1.4\varphi$$ (7)

Where:

*F*_*cd*_*F*_*qd*_*F*_*γd*_ = depth factors

Meyerhof [63] the depth factors can be expressed as:

$$for\;\varphi \geq 10^{0}$$

$$F_{cd}=1+0.2\frac{D_{f}}{B}tan\;(45+\frac{\varphi }{2})$$ (8)

$$F_{qd}=F_{\gamma d}=1+0.1\frac{D_{f}}{B}tan\;(45+\frac{\varphi }{2})$$ (9)

By using the above relationships, the theoretical ultimate bearing capacities of unreinforced soils can be calculated.

### Ultimate bearing capacity of reinforced soil foundation

In this study, a new bearing capacity formula, which was developed by Chen and Abu-Farsakh [17] to estimate the ultimate bearing capacity of reinforced soil foundations, was adopted. This method considers both confinement and membrane effects of the reinforcements on the increase in the ultimate bearing capacity. A limit equilibrium stability analysis of RSFs was performed based on the proposed failure mechanism. In this new method, they considered the failure mechanism based on the previous studies of Chen [34] and the punching shear failure followed by a general shear failure. The corresponding formulae can be expressed as follows:

$$q_{u(R)}=q_{u(UR)}+{\Delta q}_{p}+{\Delta q}_{t}$$ (10)

$$q_{u(UR)}={cN}_{C}+\gamma (D_{f}+D_{p})N_{q}+\frac{1}{2}\gamma BN_{\gamma }$$ (11)

$${\Delta q}_{p}=\frac{2c_{a}D_{p}}{B}+\gamma {D_{p}}^{2}(1+\frac{2D_{f}}{D_{p}})\frac{K_{s}tan\varphi }{B}-\gamma D_{p}$$ (12)

$${\Delta q}_{t}=\sum_{i=1}^{N_{p}}\left(\frac{2T_{ix}tan\delta +2T_{i}sin\alpha }{B}\right)+\sum_{i=N_{p}+1}^{N}\left(\frac{{4T}_{ix}(u+(i-1)h-D_{p})}{B^{2}}\right)+\sum_{i=N_{p}+1}^{N_{T}}\left(\frac{{2T}_{i}sin\xi }{B}\right)$$ (13)

$$T_{ix}=\left[\begin{array}{l} T_{i}\cos\alpha \;i\leq N_{p}\\ \frac{T_{i}sin(\frac{\pi }{4}+\frac{\varphi }{2}+\beta -\xi )}{sin(\frac{\pi }{4}+\frac{\varphi }{2}+\beta )}\;i>N_{p} \end{array}\right]$$ (14)

$$\beta =\left[\begin{array}{l} \;0\;u+(i-1)h\leq D_{p}+\frac{B}{2}tan(\frac{\pi }{4}+\frac{\varphi }{2})\\ \theta \;u+(i-1)h\leq D_{p}+\frac{B}{2}tan(\frac{\pi }{4}+\frac{\varphi }{2}),\;r_{0}e^{\theta tan\varphi }=\frac{u+(i-1)h}{cos(\frac{\pi }{4}-\frac{\varphi }{2}-\theta )} \end{array}\right]$$ (15)

By applying the above relationships, the theoretical ultimate bearing capacity of the reinforced soil foundation can be calculated.

## Results and discussions

The results obtained from Plaxis to determine the ultimate bearing capacity and the settlement of the footing were the load settlement curves of the reinforced and unreinforced soils of the three mentioned sites, while, the results obtained from the analytical analysis by Meyerhof [63] equation and the method derived by Chen and Abu-Farsakh [17] were the BCR values of these soils with geogrid reinforcement.

### Unreinforced soils

**Fig 6:**
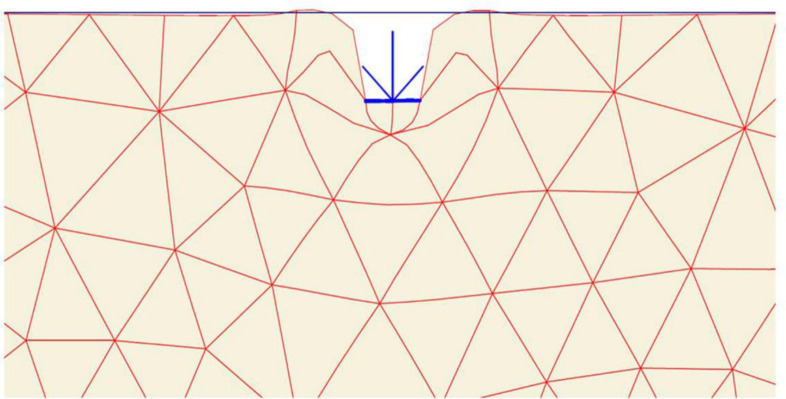
Deformed mesh of unreinforced soil under the application of the failure load.

**Fig 7:**
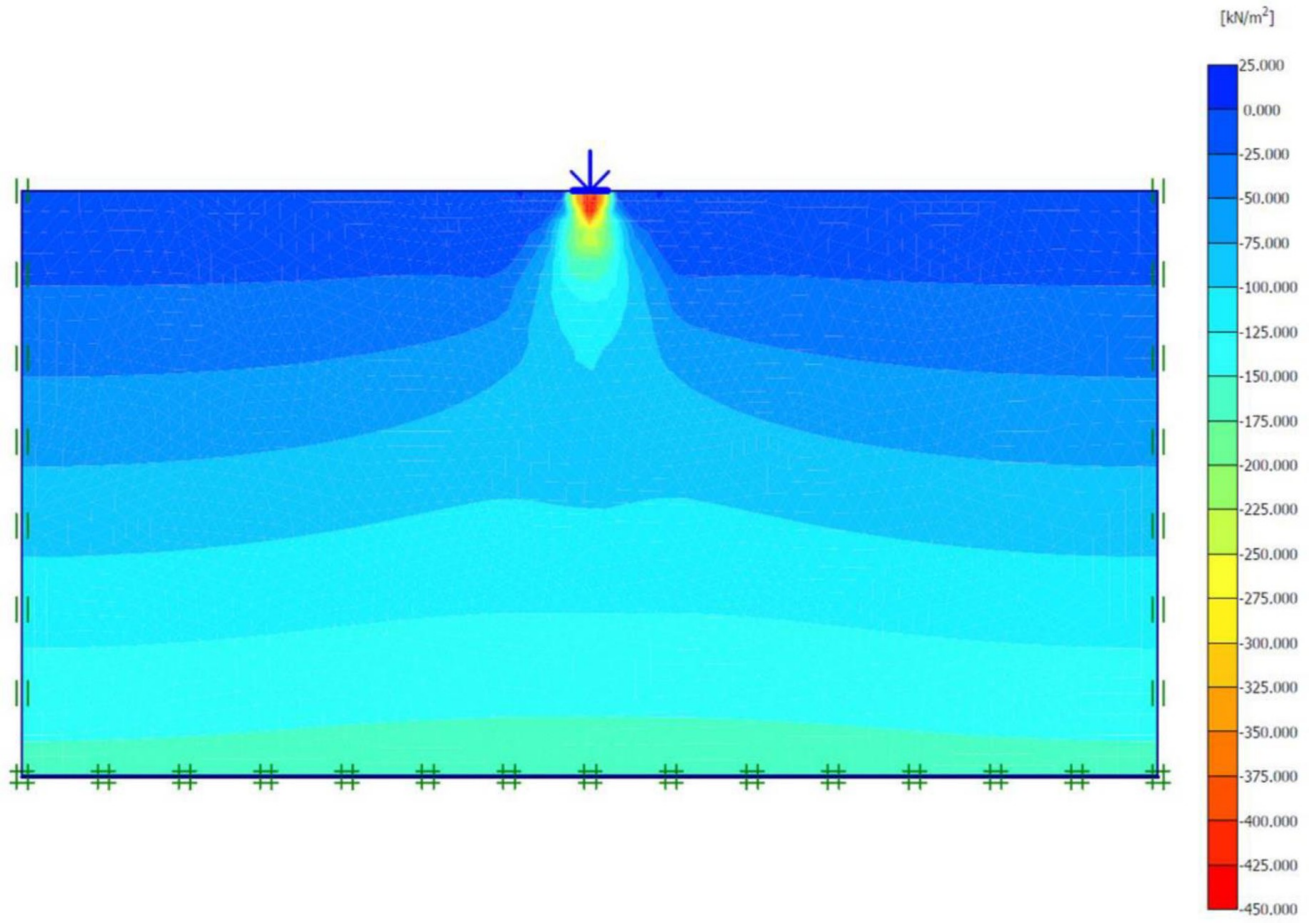
Vertical effective stress generated within unreinforced soil due to the failure load application.

**Fig 8:**
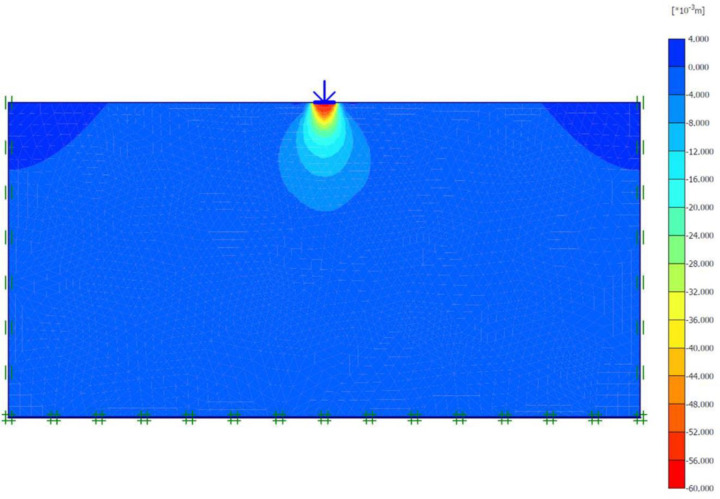
Vertical displacement generated within unreinforced soil due to the failure load application.

**Fig 9:**
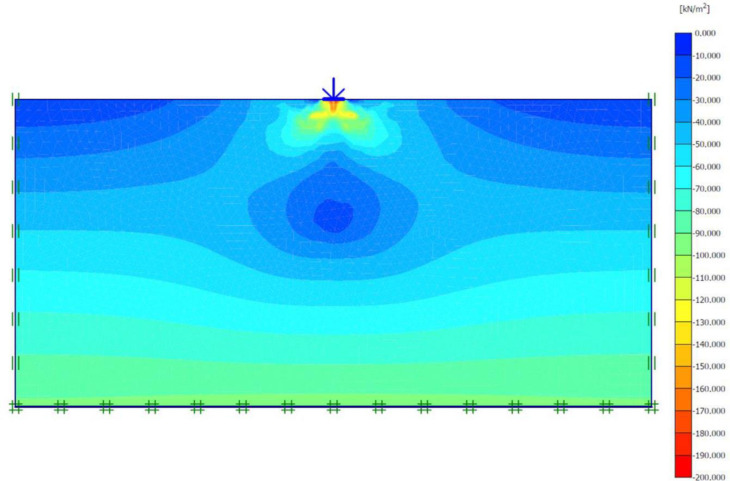
Horizontal effective stresses generated within unreinforced soil due to the failure load application.

**Fig 10:**
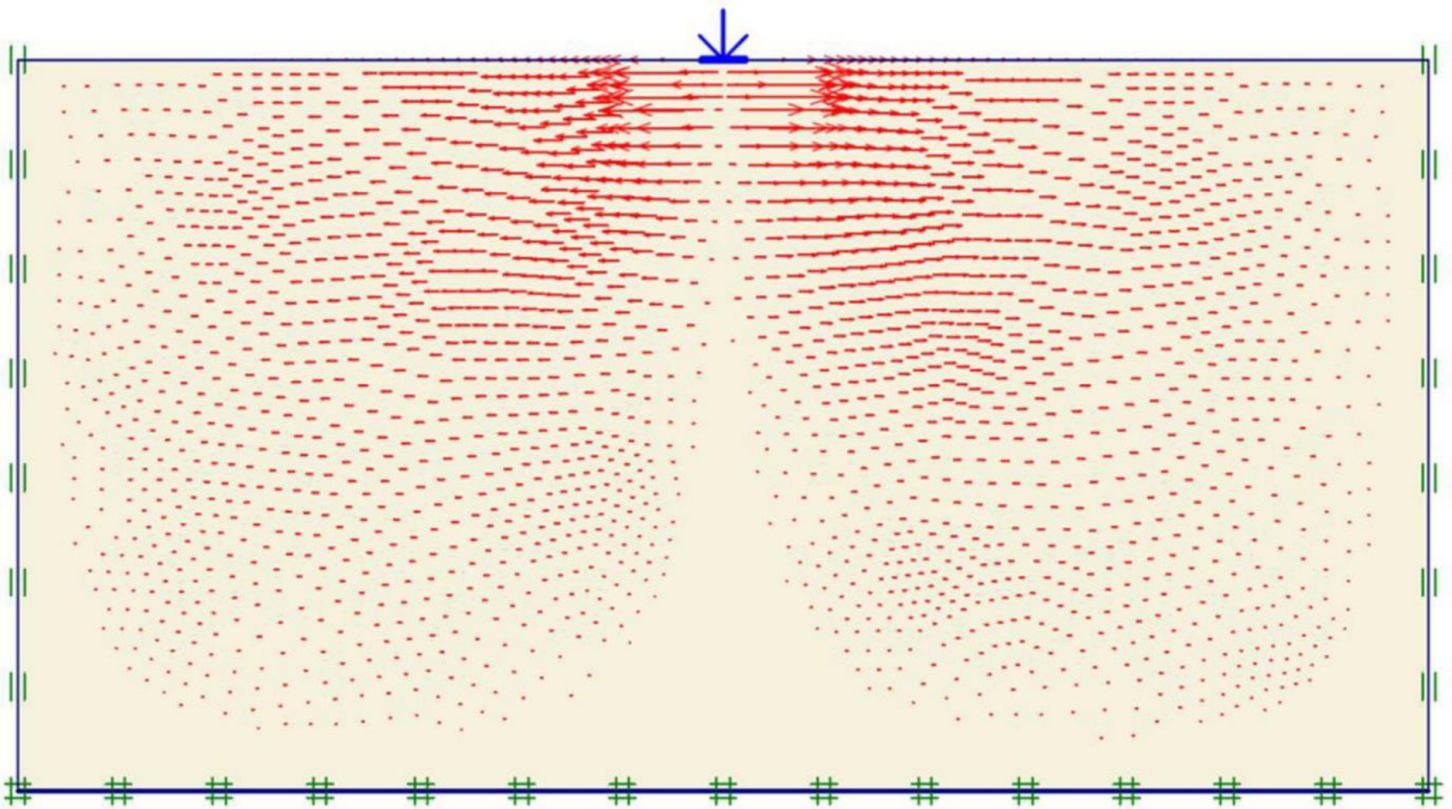
Horizontal displacement generated within the unreinforced soil due to the failure load application.

Three FEM simulations were conducted using the Plaxis software to evaluate the ultimate bearing capacity of unreinforced soil for each site. Fig 6 shows the deformed mesh (scaled up to 15 times) of the soil under the application of the failure load. From Fig 6, a small soil heave at the edges of the footing and a settlement of 57.43 mm can be seen, which indicated the shear failure of the soil. Figs 7 and 8 show the developed vertical stress and vertical displacement of the unreinforced soil, respectively, under the application of the failure load. Figs 7 and 8 show the bubble of the vertical stress and the vertical displacement increments, respectively, within the soil profile due to the application of strip loading [64]. However, the vertical stress and the vertical displacement decreased with an increase in the depth, as shown in these figures by the contours shading values. The corresponding stresses and displacements in the horizontal direction are presented in Figs 9 and 10, respectively. The maximum horizontal stresses in Fig 9 were concentrated directly under the footing within a depth of *B* and horizontally with a width of *B*; in addition, it was clear from the horizontal stresses shading that the soil failed under the local shear.

**Fig 11:**
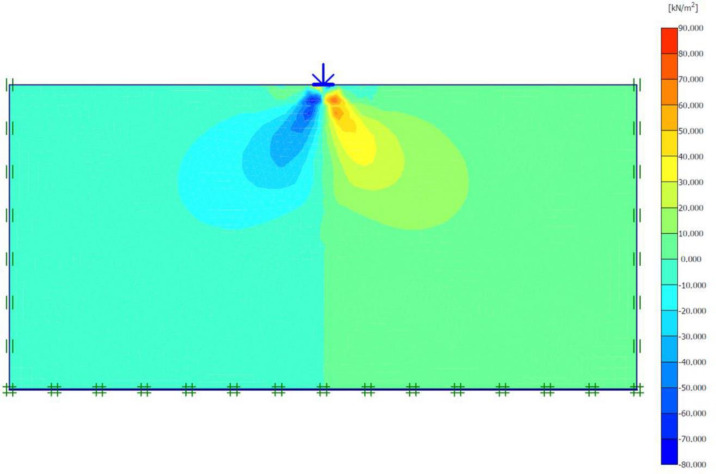
Shear stresses generated within the unreinforced soil due to the failure load application.

**Fig 12:**
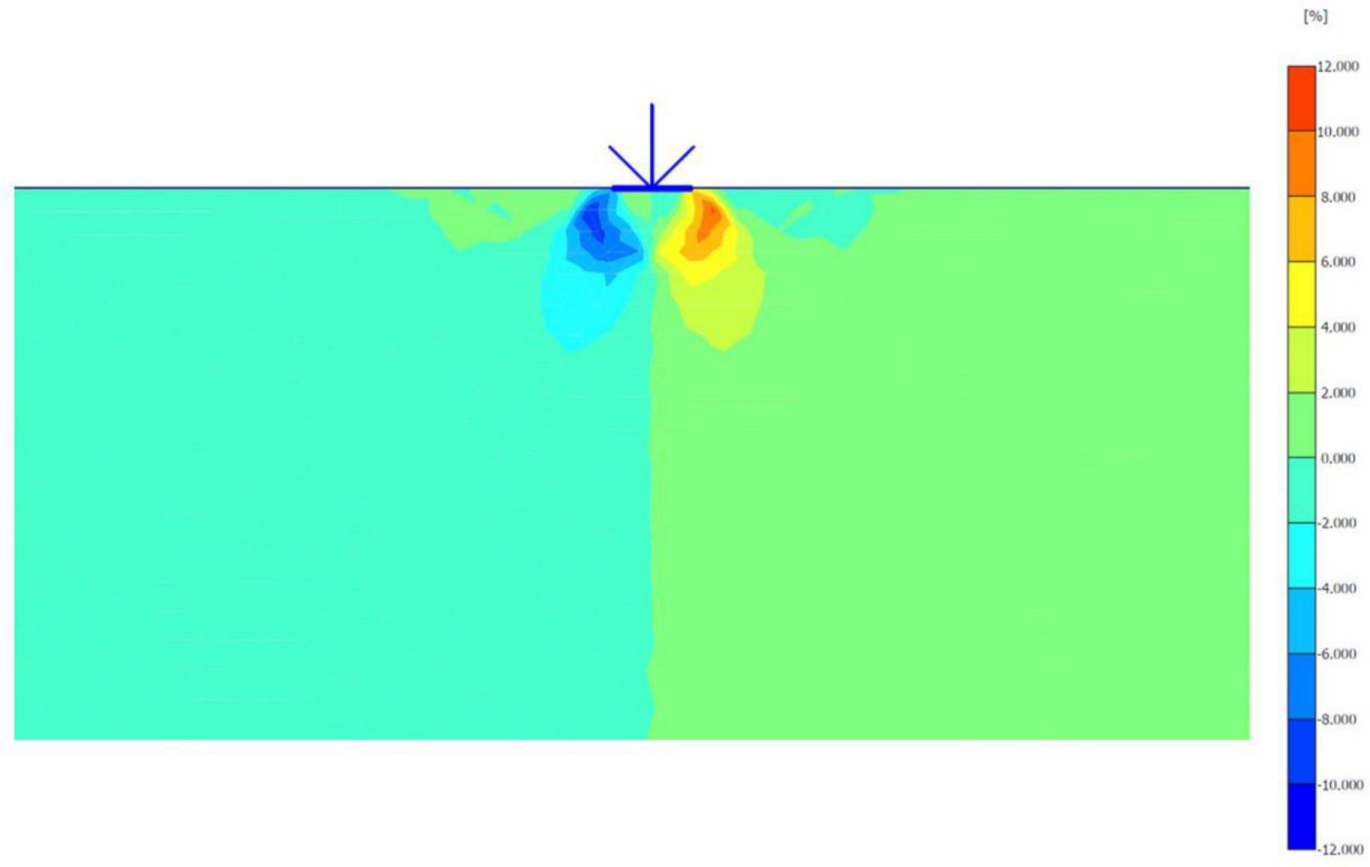
Shear strains generated within the unreinforced soil due to the failure load application.

**Fig 13:**
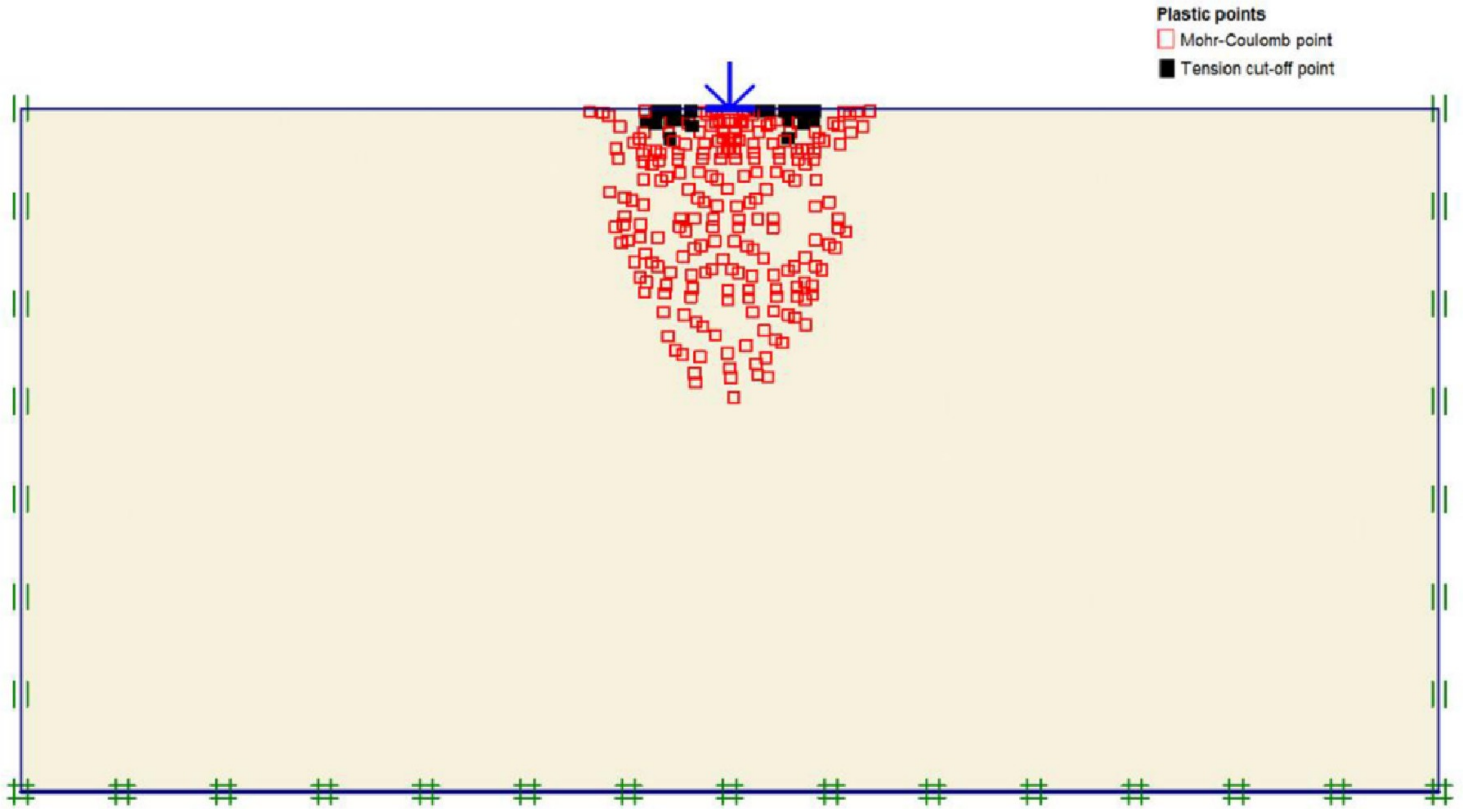
Plastic and tension points generated within the unreinforced soil due to the failure load application.

The maximum portion of the horizontal displacement presented in Fig 10 was located near the soil surface, and this was the reason for the soil heave at the footing edges. However, these horizontal stresses and displacements significantly affected the geogrid behaviour, as discussed later in the reinforced soil section. The shear stresses and strains associated with the failure are depicted in Figs 11 and 12, respectively. Note that the maximum shear stresses and strains or the highly sheared zone were located under the edges of the footing and almost propagated within a depth of 2*B*, horizontally with a distance of *B* from the footing edges, and decreased significantly at the lower depths. However, the local shear failure was almost obvious from the shadings of the shear stresses shown in Fig 11. Fig 13 presents the plastic points or the failure plastic points generated within the soil mass under the application of the failure load. A plastic point is a point corresponding to the irreversible stress and deformation which is located on the Mohr-Coulomb failure envelope (the envelope is a function of the internal friction angle of the soil cohesion).

Fig 13 also shows the tension points (points with black colour) at the soil surface, which corresponded to the tension cracks (tension stress areas). However, these tension points indicated that the soil failed under tension instead of shear. The theoretical ultimate bearing capacity of the unreinforced soil was obtained by applying Eqs (4)–(9). The shear strength parameters (c and *φ*) and the unit weight (*γ*) used in the following equations are shown in Table 3.

Al-Hamedat site:

$N_{q}={tan}^{2}(45+\frac{20}{2})e^{\pi \;tan20}=6.4$

$N_{c}=cot20(6.4-1)=14.83$

$N_{\gamma }=(6.4-1)tan1.4*20=5.39$

$F_{cd}F_{qd}F_{\gamma d}=1\;as\;the\;footing\;depth\;(D_{f}=0)$

$q_{u}=40*14.83*1+0+0.5*17*.6*5.39*1=620\;{KN/m}^{2}$

Ba’shiqah site:

$N_{q}={tan}^{2}(45+\frac{25}{2})e^{\pi \;tan25}=10.66$

$N_{c}=cot25(10.66-1)=20.72$

$N_{\gamma }=(10.66-1)tan1.4*25=10.88$

$F_{cd}F_{qd}F_{\gamma d}=1\;as\;the\;footing\;depth\;(D_{f}=0)$

$q_{u}=15*20.72*1+0+0.5*15*.6*10.88*1=359\;{KN/m}^{2}$

Al-Rashidia site:

$N_{q}={tan}^{2}(45+\frac{28}{2})e^{\pi \;tan28}=17.81$

$N_{c}=cot25(10.66-1)=31.61$

$N_{\gamma }=(10.66-1)tan1.4*25=13.7$

$F_{cd}F_{qd}F_{\gamma d}=1\;as\;the\;footing\;depth\;(D_{f}=0)$

$q_{u}=0*31.61*1+0+0.5*16*.6*13.7*1=65\;{KN/m}^{2}$

**Fig 14:**
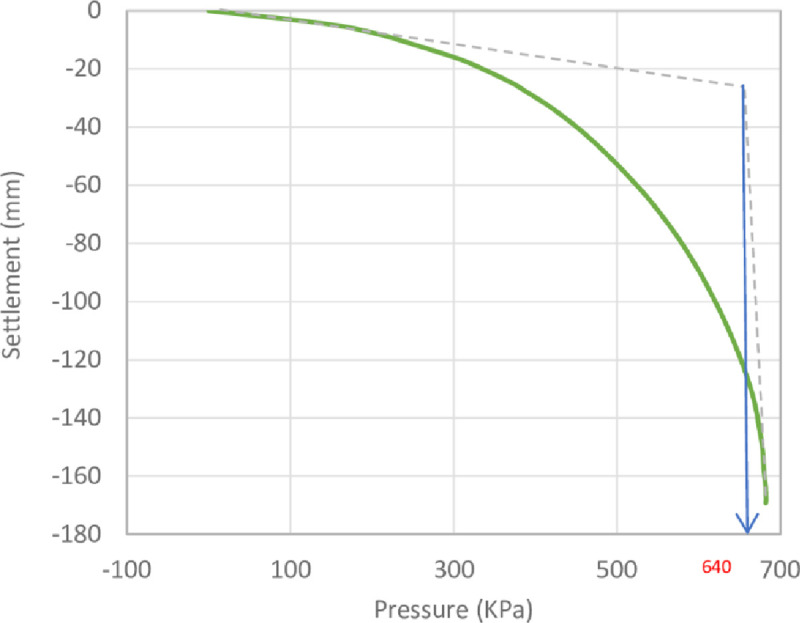
Pressure–settlement curve and determination of ultimate bearing capacity of Al-Hamedat site.

**Fig 15:**
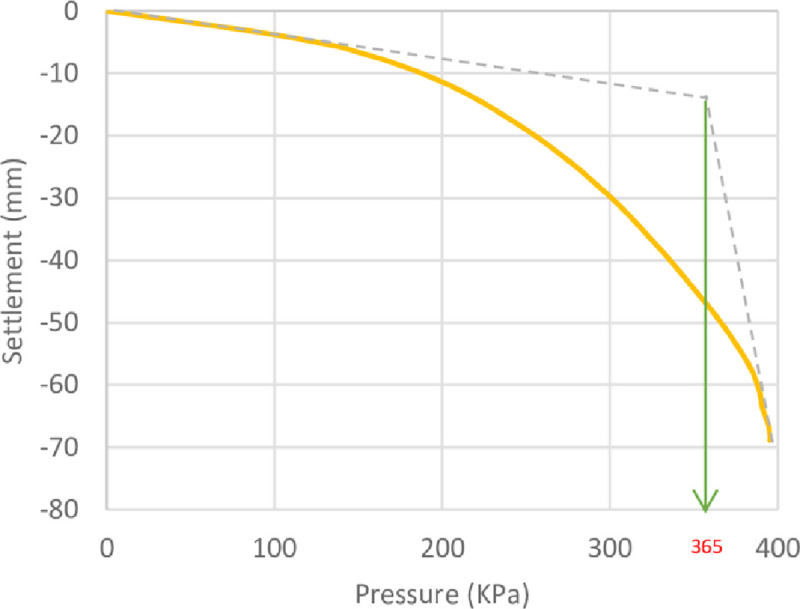
Pressure–settlement curve and determination of ultimate bearing capacity of Al-Rashidia site.

**Fig 16:**
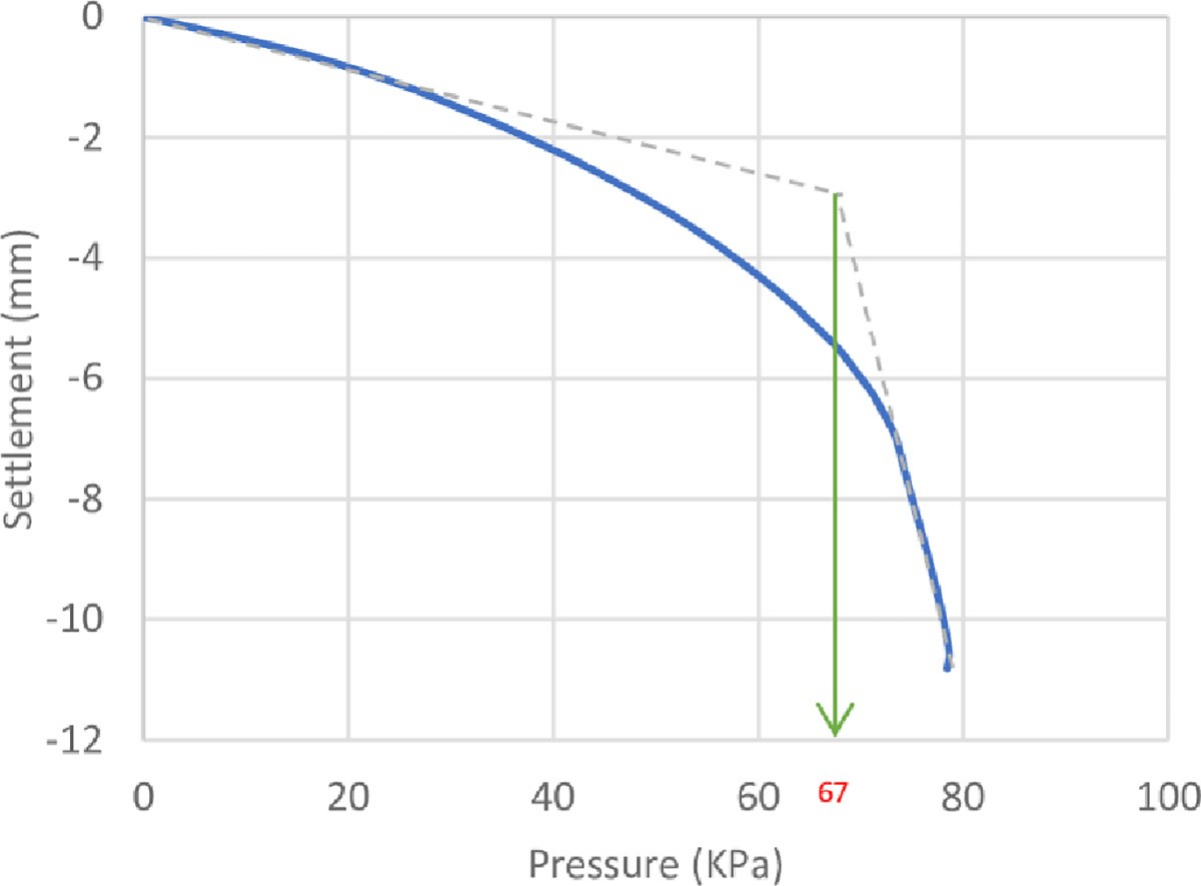
Pressure–settlement curve and determination of ultimate bearing capacity of Ba’shiqa site.

The results of the unreinforced soil foundations obtained by the numerical analysis and the theoretical ultimate bearing capacity derived by Meyerhof [63] are shown in Table 6. Here, it can be seen that the numerical bearing capacity values were greater than the theoretical values. The high value of the bearing capacity might be attributed to the fact that the bearing capacity equations usually underestimated (more conservative) the ultimate bearing capacity of the soil [64]. The pressure–settlement curves from the numerical analysis of the unreinforced soil foundations of the three sites are shown in Figs 14–16. Moreover, these figures show the method used for determining the ultimate bearing capacity from the load–settlement curves; it represents the conservative and the most real state of failure. This method is the tangent intersection method, developed by Trautmann and Kulhawy [65].

**Table 6:** Numerical and theoretical ultimate bearing capacity of the three sites’ soils.

Site	Numerical (*q*_*u*_ *kN*/*m*^2^)	Theoretical (*q*_*u*_ *kN*/*m*^2^)
Al-Hamedat	640	620
Ba’shiqa	365	359
Al-Rashidia	67	65

From Figs 14 to 16, it can be noticed that Al-Hamedat’s soil shows higher bearing capacity (*q*_*u*_ = 640 *kPa*) than the other two sites where Ba’shiqah soil shows the intermediate bearing capacity value (*q*_*u*_ = 365 *kPa*) and Al-Rashidia soil presents the lowest (*q*_*u*_ = 67 *kPa*) among the soils. This difference can be due to the soil characteristics and properties as indicated in Table 3 and S1 Table. It is referred that the soil of Al-Hamedat site is hard clay with high cohesion (*c* = 40 *kPa*), Al-Rashidia is sandy soil with high friction angle (*φ* = 28°) with zero cohesion (c = 0 KPa) while, the soil of Ba’shiqa site is classified as low to medium clay with relatively low cohesion (*c* = 15 *KPa*) in comparison to Al-Hamedat’s soil.

### Reinforced soils

**Fig 17:**
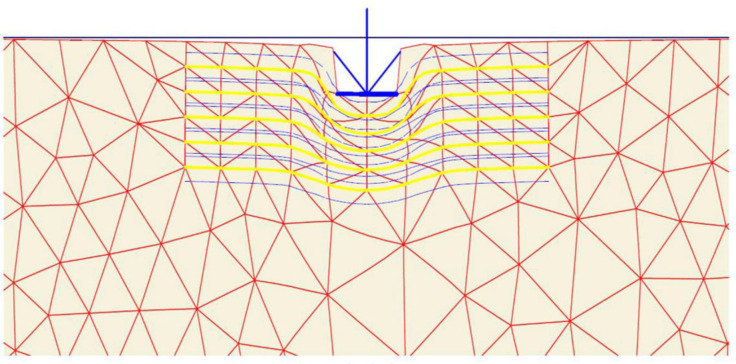
Deformed mesh of the geogrid reinforced soil.

**Fig 18:**
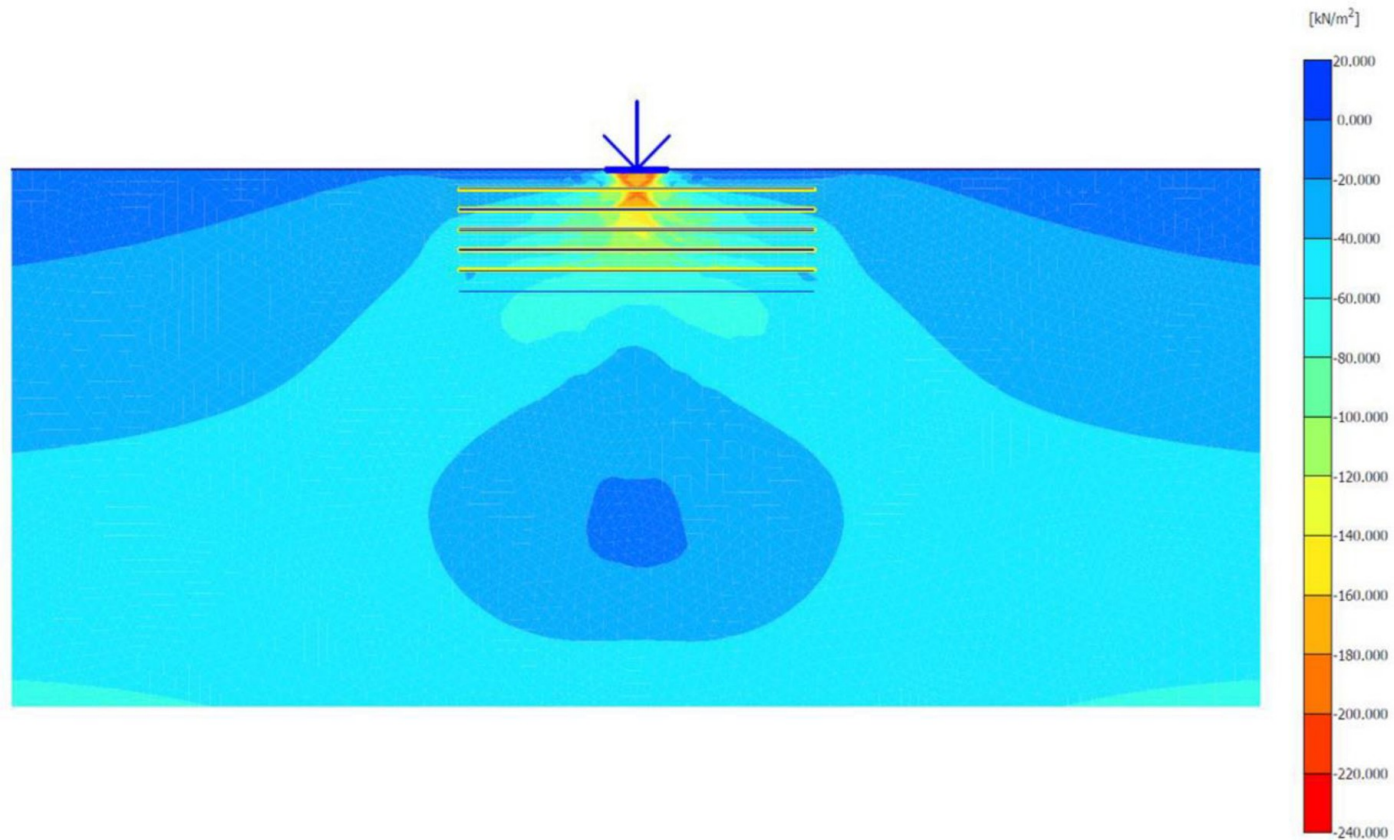
The horizontal effective stress generated within the geogrid reinforced soil.

**Fig 19:**
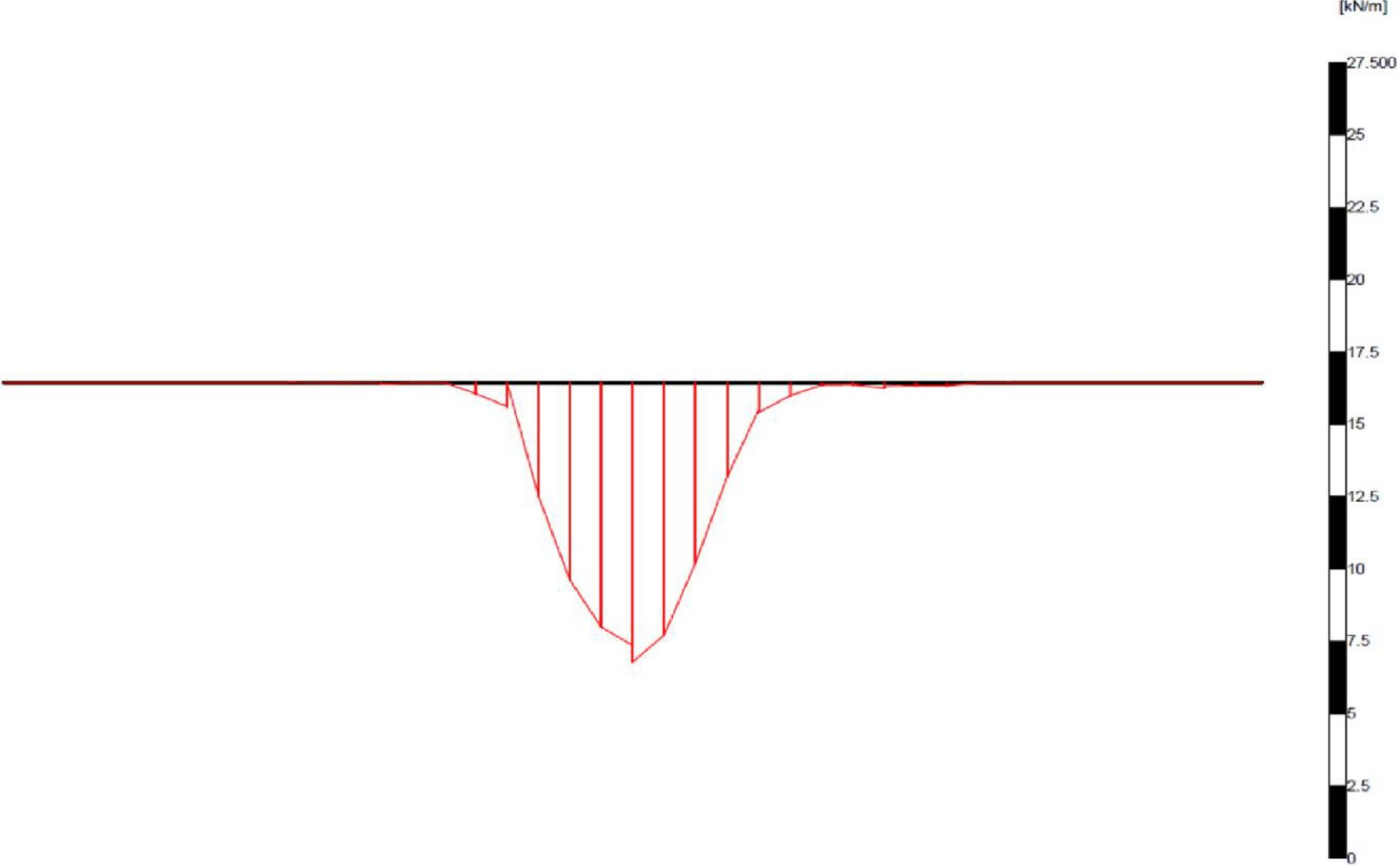
The axial force within the geogrid reinforcement.

Ninety FEM simulations were conducted on reinforced soil foundations to examine the effect of geogrid reinforcement on the ultimate bearing capacity and settlement of the strip footing located at the three mentioned sites. The deformed mesh (scaled up to 10 times) of the geogrid reinforced soil is shown in Fig 17. Moreover, the settlement was reduced to 44.68 mm by the inclusion of the geogrid reinforcement, where the reduction in the settlement was attributed to the uplift forces generated by the geogrid reinforcement during the deformation and the mobilisation of the axial tensile forces of the reinforcement layers. Furthermore, the soil heave at the edges of footing already disappeared, which implied that the soil did not fail under shear, as the unreinforced soil mentioned earlier. Fig 18 shows the horizontal stresses generated within the reinforced soil mass. It can be seen that the horizontal stresses were slightly increased to a value of 228.96 kN/m^2^ due to the transfer of a part of the vertical load to a horizontal load carried by the reinforcement and in turn to the surrounding soil. Moreover, the horizontal stresses were distributed along the layers of reinforcement to a width of 5*B*, which indicated the interlocking and the interaction of soil and geogrid layers; as a result, the tensile forces within the reinforcement were mobilised as shown in Fig 19.

**Fig 20:**
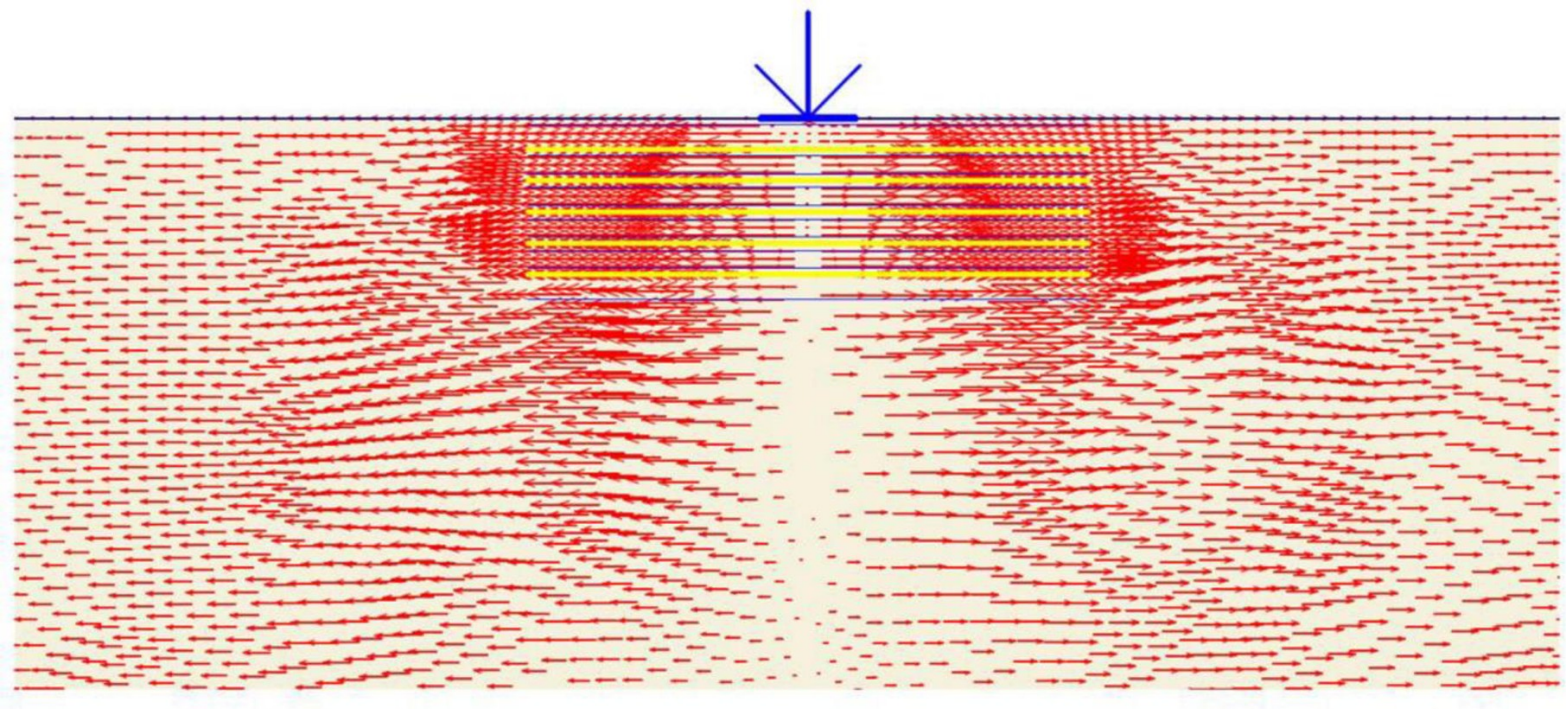
The horizontal displacement generated within the geogrid reinforced soil.

**Fig 21:**
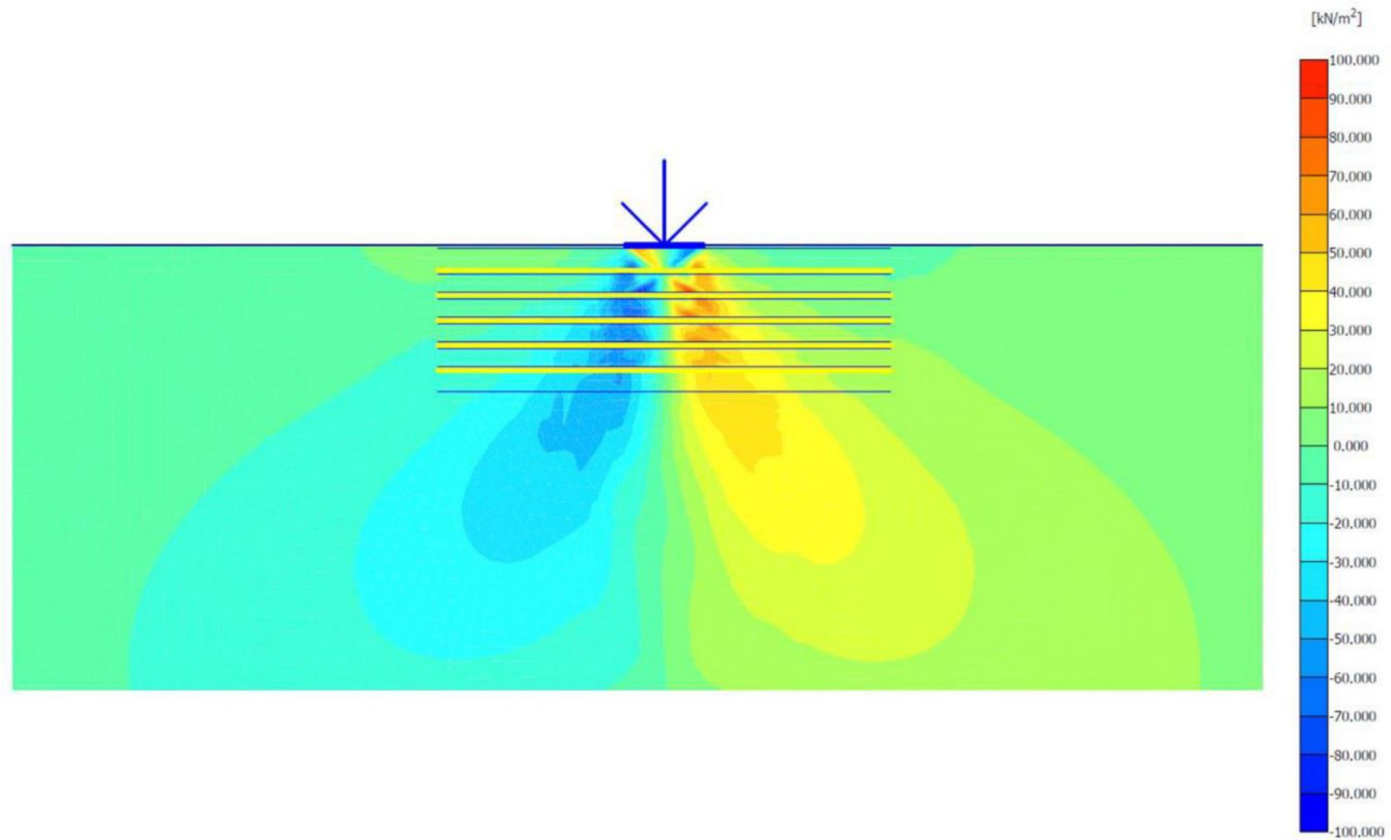
The shear stress generated within the geogrid reinforced soil.

**Fig 22:**
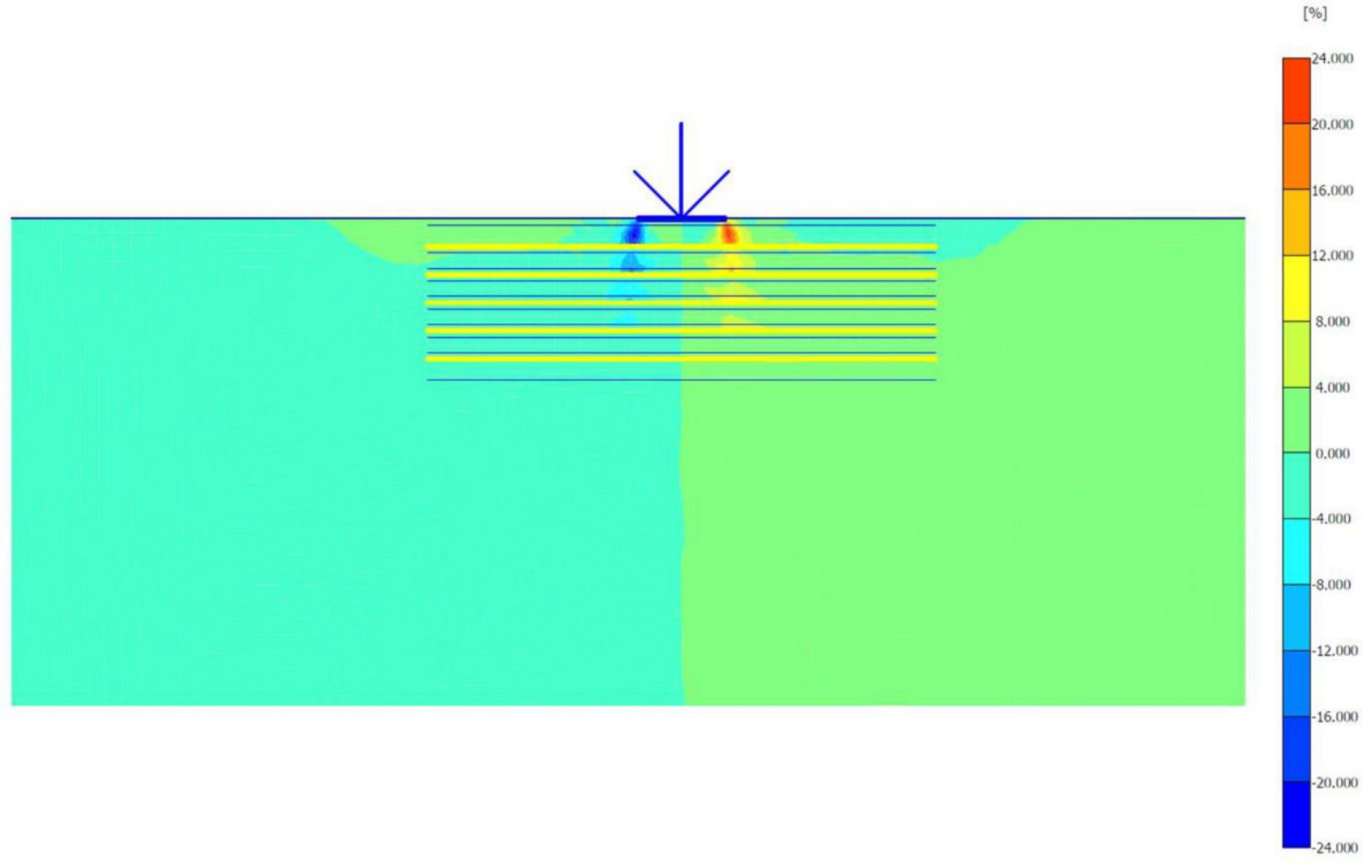
The shear strain generated within the geogrid reinforced soil.

**Fig 23:**
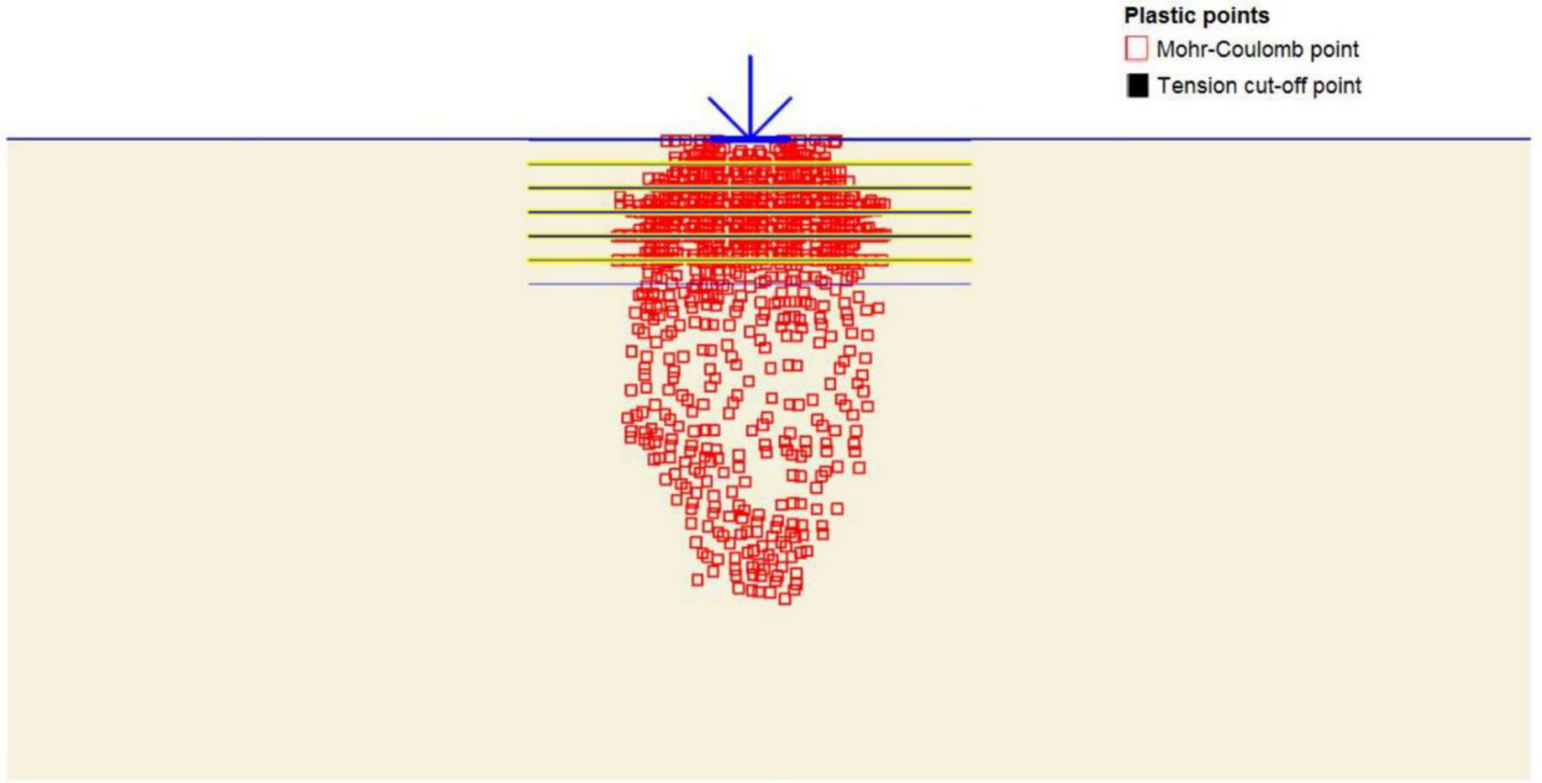
Plastic points generated within the geogrid reinforced soil under load application.

Fig 20 depicts the horizontal displacement distribution within reinforced soil. It is clear that displacement is reduced to 8.68 mm due to the reinforcement layers restriction, the arrows are almost equally distributed along the reinforcement layers and small displacement values induced at the soil surface in comparison to the unreinforced state where most of the horizontal displacement occurred at the upper part of the soil causing the soil heave. Consequently, the shear failure of the soil is prevented by transferring the applied vertical load to tension forces in the geogrid reinforcement by the skin friction and bearing between soil and reinforcement. Figs 21 and 22 show the shear stresses and strains of reinforced soil and their distribution along the geogrid reinforcement, respectively. It is noticed that areas of shear stresses and strains concentration under the foundation are reduced through the distribution of stresses and strains along and through the reinforcement layers which results in changing of the failure plane and prevent the failure within the reinforced zone. The plastic points within the reinforced zone are depicted in Fig 23. It is shown that plastic points are heavily concentrated along the reinforced zone which indicates the extreme stresses generated at the interface between the soil and geogrid. Consequently, this justifies the interaction between soil and geogrids, and the alteration of the failure mechanism.

### Effect of geogrid width *(b)* and number of geogrid layer *(N)* on ultimate bearing capacity

**Fig 24:**
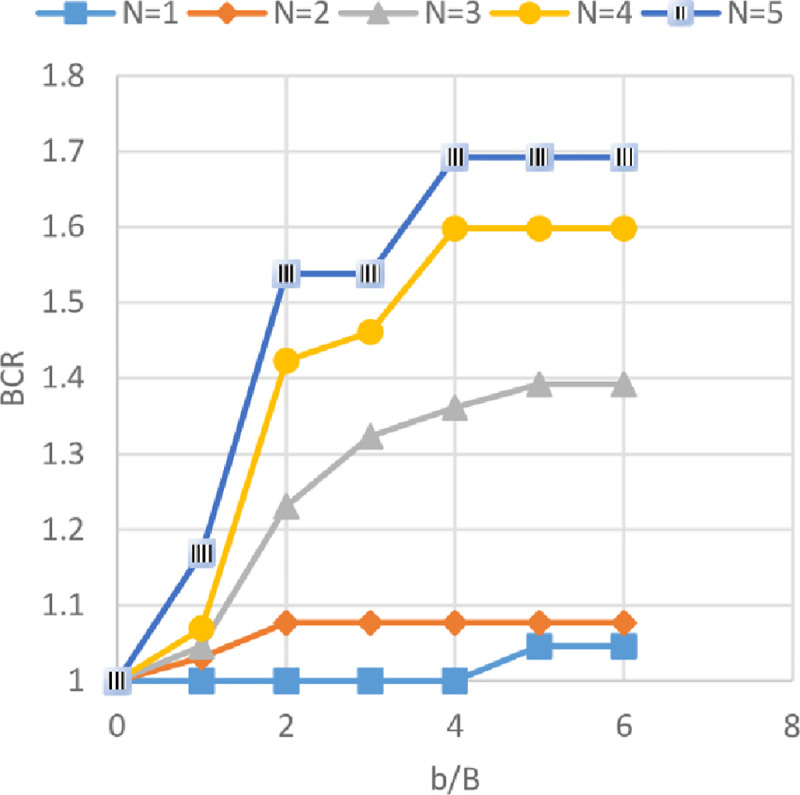
BCR vs b/B with different geogrid number (*N*) for Al-Hamedat site.

**Fig 25:**
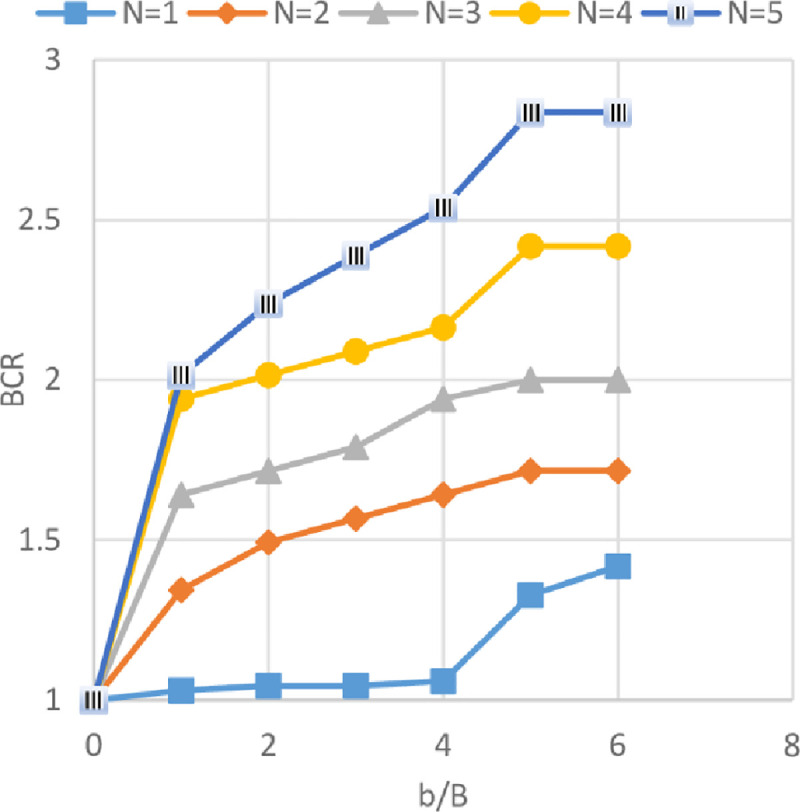
BCR vs b/B with different geogrid number (*N*) for Al-Rashidia site.

**Fig 26:**
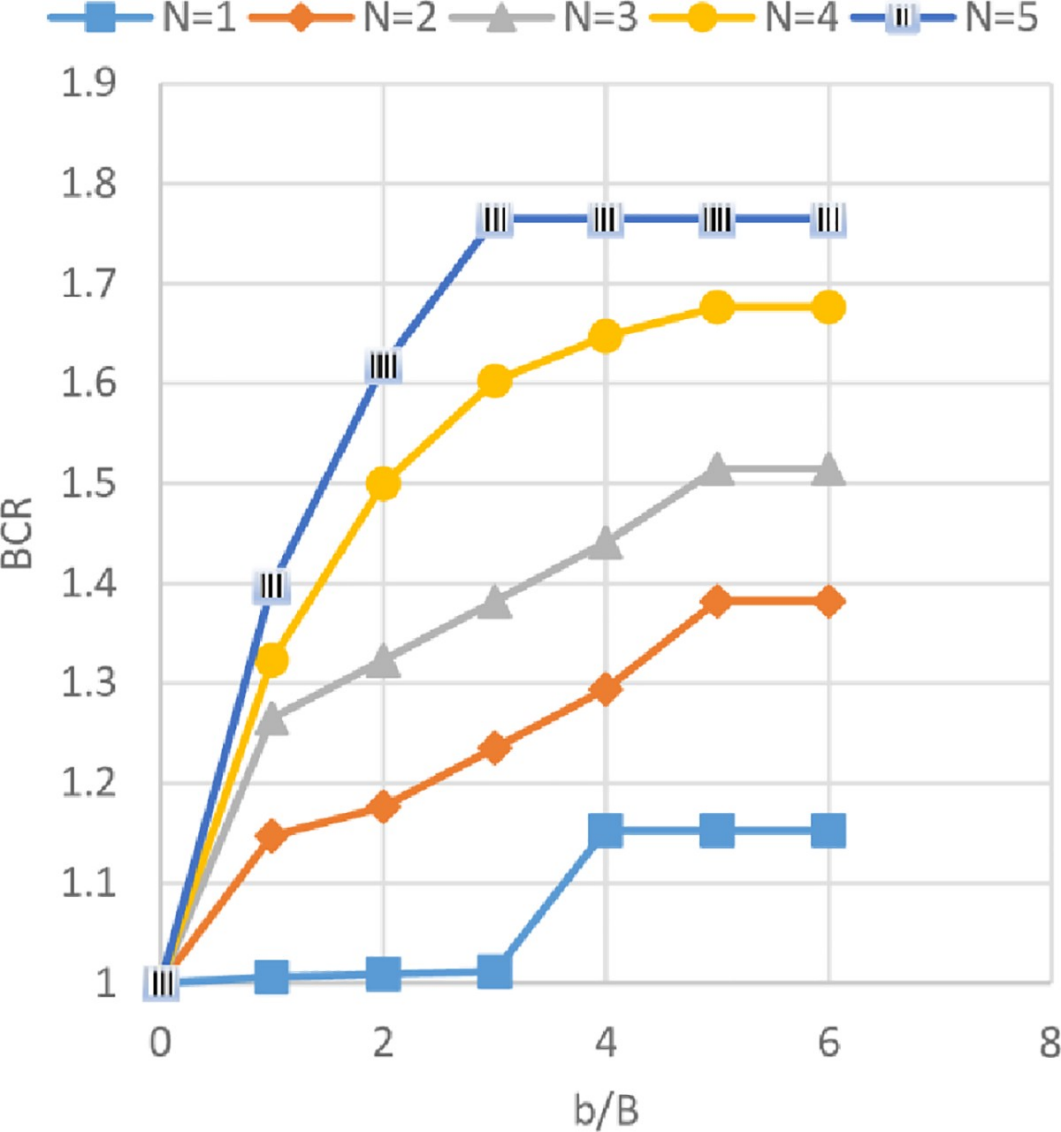
BCR vs b/B with different geogrid number (*N*) for Ba’shiqa site.

Figs 24–26 show the variation of BCR with six different geogrid widths *(b)* for 1 to 5 number of geogrid layers (*N*) for the three sites Al-Hamedat, Al-Rashidia, and Ba’shiqa, respectively. From Figs 24 to 26, it can be seen that the increased width of geogrid *(b)* and geogrid number *(N)* leads to an increase of the BCR for all three sites. Furthermore, the soil at Al-Rashidia promotes higher improvement of the ultimate bearing capacity than the other two sites. The improvement can be due to the difference of the soil properties and grain size as presented in Table 3 and S1 Table. Al-Rashidia soil is sandy and has a friction angle (*φ* = 28°) greater than the other two sites in which, the passive and frictional forces between soil and geogrid will be higher than the two clayey sites [8]. For Al-Hamedat and Ba’shiqa sites with clayey soils, the soil of Ba’shiqa site which is low to medium clay shows better improvement than the soil of Al-Hamedat site which is hard clay in terms of ultimate bearing capacity. Hence, by using geogrid reinforcement with weak clay, the soil may improve to stiffer clay. However, the maximum improvement in the ultimate bearing capacity can be obtained at *b/B* = 5 for any geogrid number in these three sites, therefore, the optimum geogrid width *(b)* for the three sites is 5*B* while there was no optimum geogrid number *(N)* obtained as *N* = 5 all the three soils show a good improvement in footing bearing capacity.

### Effect of geogrid width *(b)* and number of geogrid layer *(N)* on footing settlement

**Fig 27:**
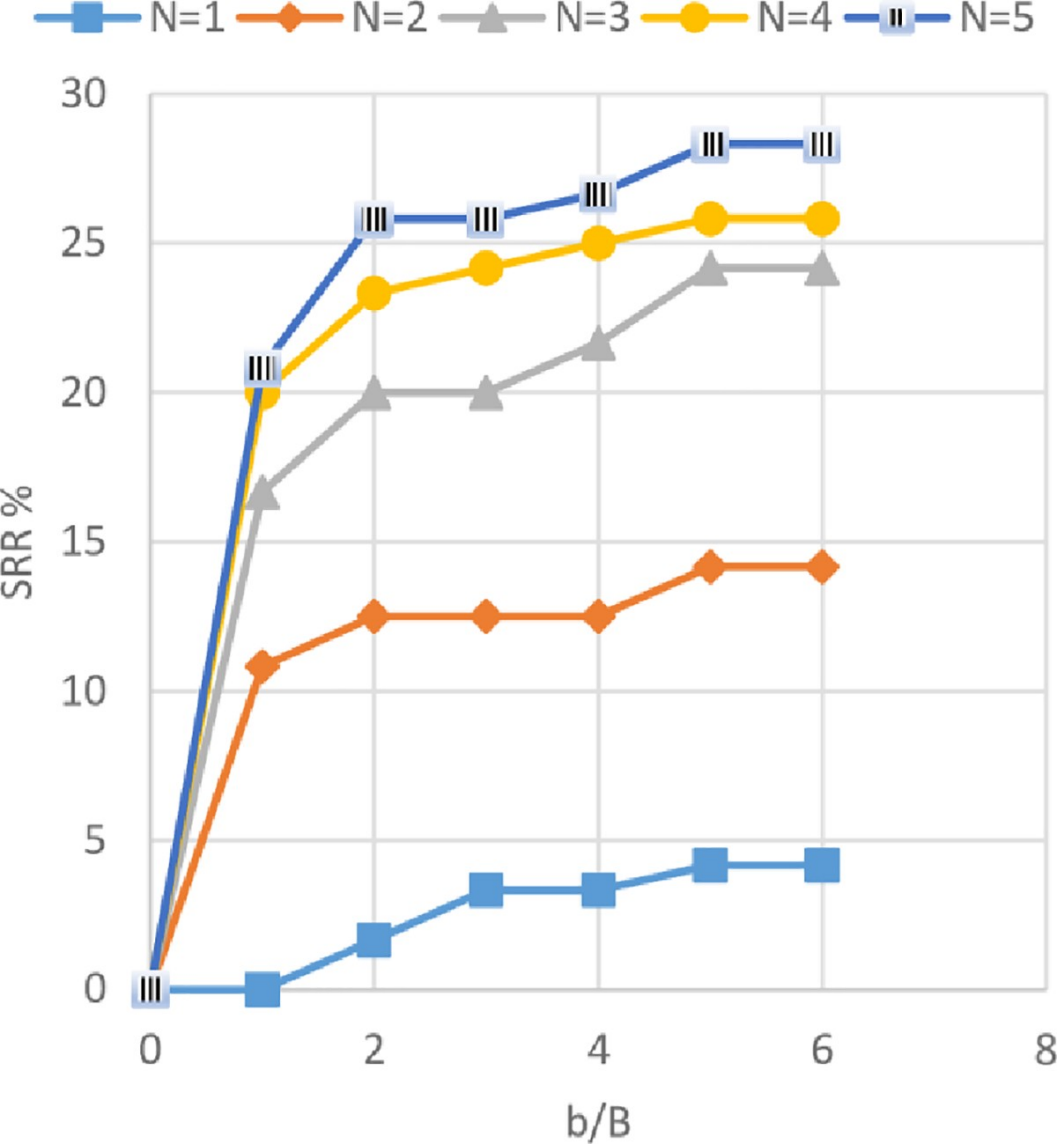
SRR vs b/B with different geogrid number (*N*) for Al-Hamedat site.

**Fig 28:**
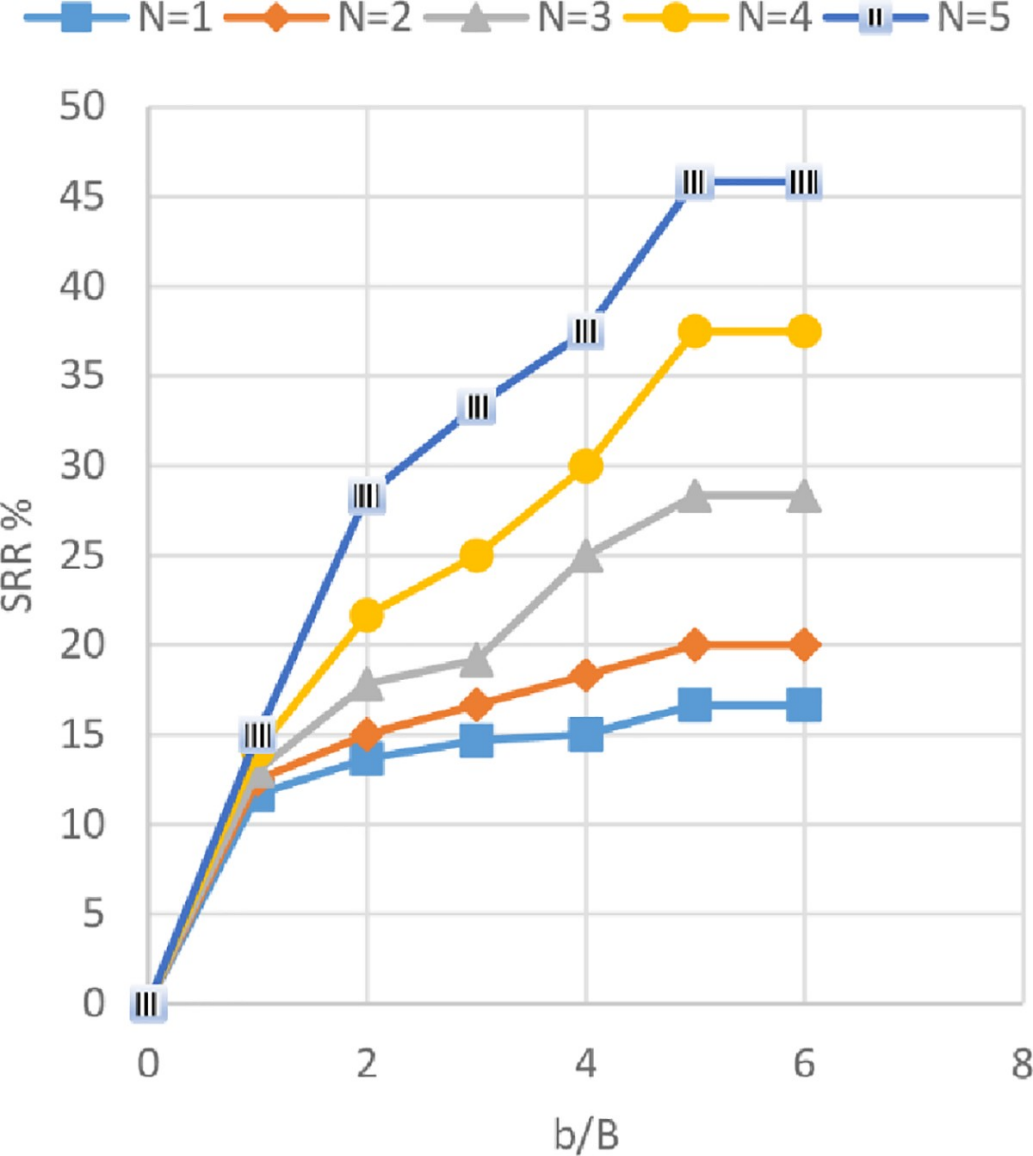
SRR vs b/B with different geogrid number (*N*) for Al-Rashidia site.

**Fig 29:**
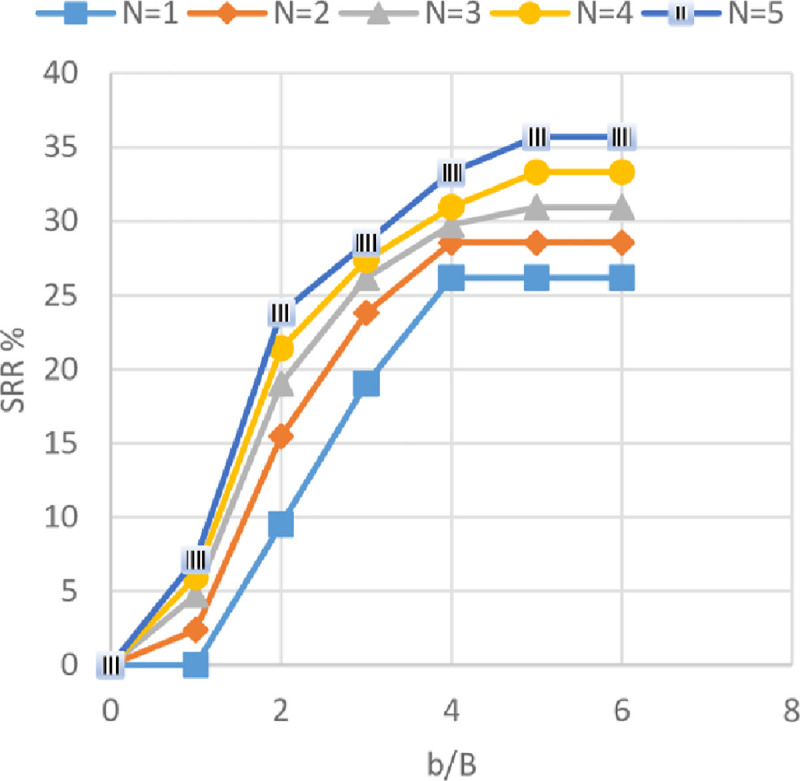
SRR vs b/B with different geogrid number (*N*) for Ba’shiqa site.

The settlement reduction ratio (SRR%) versus different geogrid widths (*b*) with 1 to 5 number of geogrid layers (*N*) is shown in Figs 27–29 for the soils of Al-Hamedat, Al-Rashidia, and Ba’shiqa sites, respectively. From these figures, it can be seen that the increase of geogrid layer width *(b)* and geogrid number (*N*) resulting in a reduction of the footing settlement for the three sites. From Figs 27 to 29, the reduction in footing settlement (SRR%) obtained from these three sites as a result of increasing the width of geogrid reinforcement *(b)* and number of geogrid layers (*N*) was observed. It is shown that a higher reduction in footing settlement as the geogrid width *(b)* increase is obtained by the soil of Ba’shiqa site for the first three geogrid layers (*N* = 1 to 3) followed by the soil of Al-Rashidia, and Al-Hamedat sites, respectively. While, at *N* = 4 and 5 the soil of Al-Rashidia started to show a higher improvement than the soil of Ba’shiqa site, in contrary to the soil of Al-Hamedat site that presents the lowest improvement.

The difference in SRR% can be due to two reasons, good friction angle of Ba’shiqa’s soil (*φ* = 25°) and the occurrence of deep footing effect [50] within the soil of Ba’shiqa site that makes the general shear failure of the soil developed below the reinforced zone. In this case, the tension of all the geogrid layers within the reinforced zone will be mobilised as the footing will fail in terms of ultimate bearing capacity after punching through the geogrid layers. The soil of Al-Rashidia site shows the second higher improvement and at N = 4 and 5, indicating a higher improvement to the footing settlement. As stated earlier, the soil of Al-Rashidia site is sandy and has the highest friction angle (*φ*) between the other two sites in which, the value of mobilised tension of the geogrid layers in the reinforced zone will be higher than that the two sites due to the sand particles interlocking with geogrid apertures. Moreover, higher frictional resistance at the contact area between the soil and the geogrid layers may occur. On the other hand, Al-Hamedat’s soil has a friction angle (*φ* = 20°) lower than that of the other two sites, which results in less friction at the soil-geogrid contacting area and less passive forces at the edges of geogrid ribs. Therefore, low improvement is portrayed on the footing settlement even though the deep-footing effect may be happening within this soil.

It can be also observed from Figs 27 to 29 that Al-Hamedat’s soil show better improvement of the footing settlement as geogrid number (*N*) increased than the geogrid width (*b*) increment while Ba’shiqa’s soil was the opposite. The increment is may be due to higher strength of the soil in Al-Hamedat site (*c* = 40 *KPa*) than Ba’shiqa’s soil (*c* = 15 *KPa*) where, it can be affected by the number of geogrid layers (*N*) more than the geogrid width (*b*). The optimum geogrid width (*b*) for the three sites at any geogrid number is also 5*B* while, there was no optimum geogrid number (*N*) obtained, the *N* = 5 all the three soils showed a good improvement of the footing settlement.

### Improvement Factor (IF)

**Fig 30:**
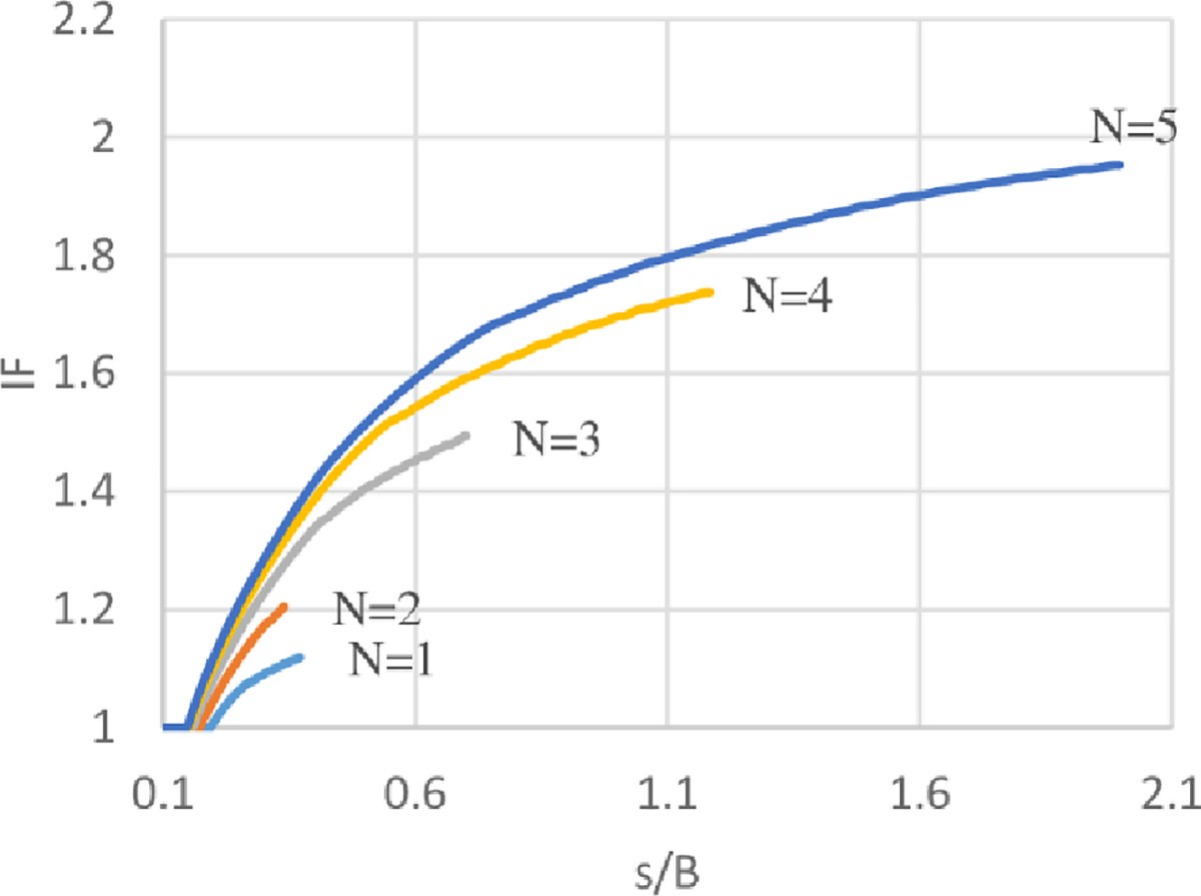
Variation of IF vs s/B with different geogrid number (*N*) for Al-Hamedat site.

**Fig 31:**
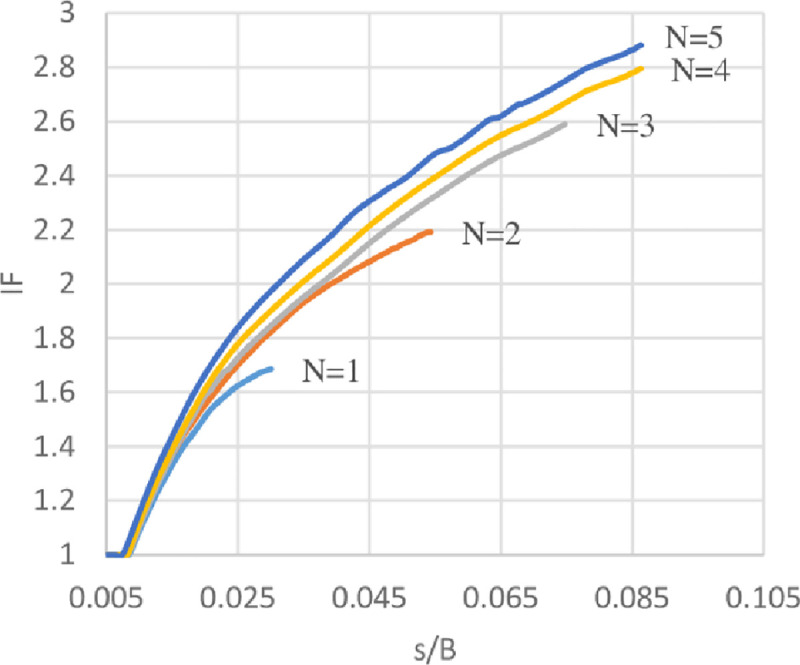
Variation of IF vs s/B with different geogrid number (*N*) for Al-Rashidia site.

**Fig 32:**
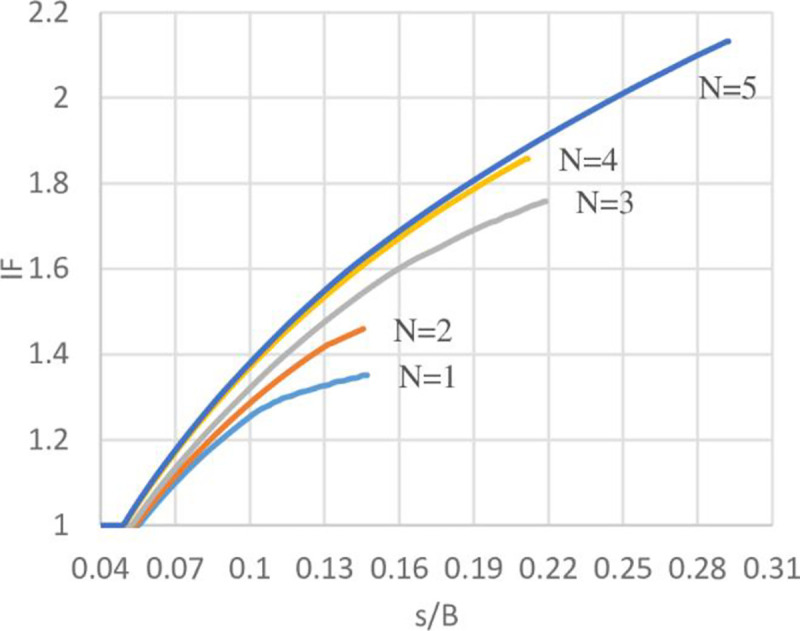
Variation of IF vs s/B with different geogrid number (*N*) for Ba’shiqa site.

Improvement factor (IF) is defined as the ratio of bearing capacity of reinforced soil (*q*_*reinforced*_) to the unreinforced soil (*q*_*unreinforced*_) at certain *s*/*B* ratios. Where *s*/*B* is the ratio of footing settlement to the footing width. IF at different *s*/*B* ratios has been calculated to compare the ultimate bearing capacity of soils with different geogrid number (*N*) at various levels of settlement. The variation of IF with *s*/*B* ratios of the three sites are shown in Figs 30–32. From these figures, it is obvious when the footing settlement increase, the improvement factor (the ultimate bearing capacity of reinforced soil) increases for any geogrid number and that is expected because the geogrid layers need the footing settlement to mobilise their tension forces, hence, improving the resistance to the applied vertical loads. It can be also noticed for the effect of geogrid number (*N*), the increasing number of geogrid layers results in IF increment, thus, reducing the initial settlement in needs to mobilise the tension of geogrid layer and make the reinforced soil to maintain resisting the applied loads even at a very high settlement without collapse.

Moreover, by using geogrid in the soil of Al-Hamedat site demonstrates less improvement factor and reaches a very large settlement to improve the footing bearing capacity in comparison with the other two sites. This large settlement is because Al-Hamedat’s soil is very strong clay (*c* = 40 KPa) with low friction angle (*φ* = 20°) than the other two sites and thus, need high settlement to mobilise the tension in geogrid layers, Ba’shiqa’s soil is also clayey (*c* = 15 KPa) with friction angle (*φ* = 25°) better than Al-Hamedat’s soil therefore, it showed better improvement in the ultimate bearing capacity and lower settlement to mobilize the tension in the geogrid layers than Al-Hamedat’s soil. While Al-Rashidia’s soil showed the highest improvement in the ultimate bearing capacity and the lowest settlement in mobilizing the tension in geogrid layers that is due to Al-Rashidia’s soil is sand with higher friction angle (*φ* = 28°) in addition, the geogrid has better performance with the sandy soil due to its friction angle and the interlocking of particles with geogrid openings.

### Comparison between numerical and analytical analysis

**Fig 33:**
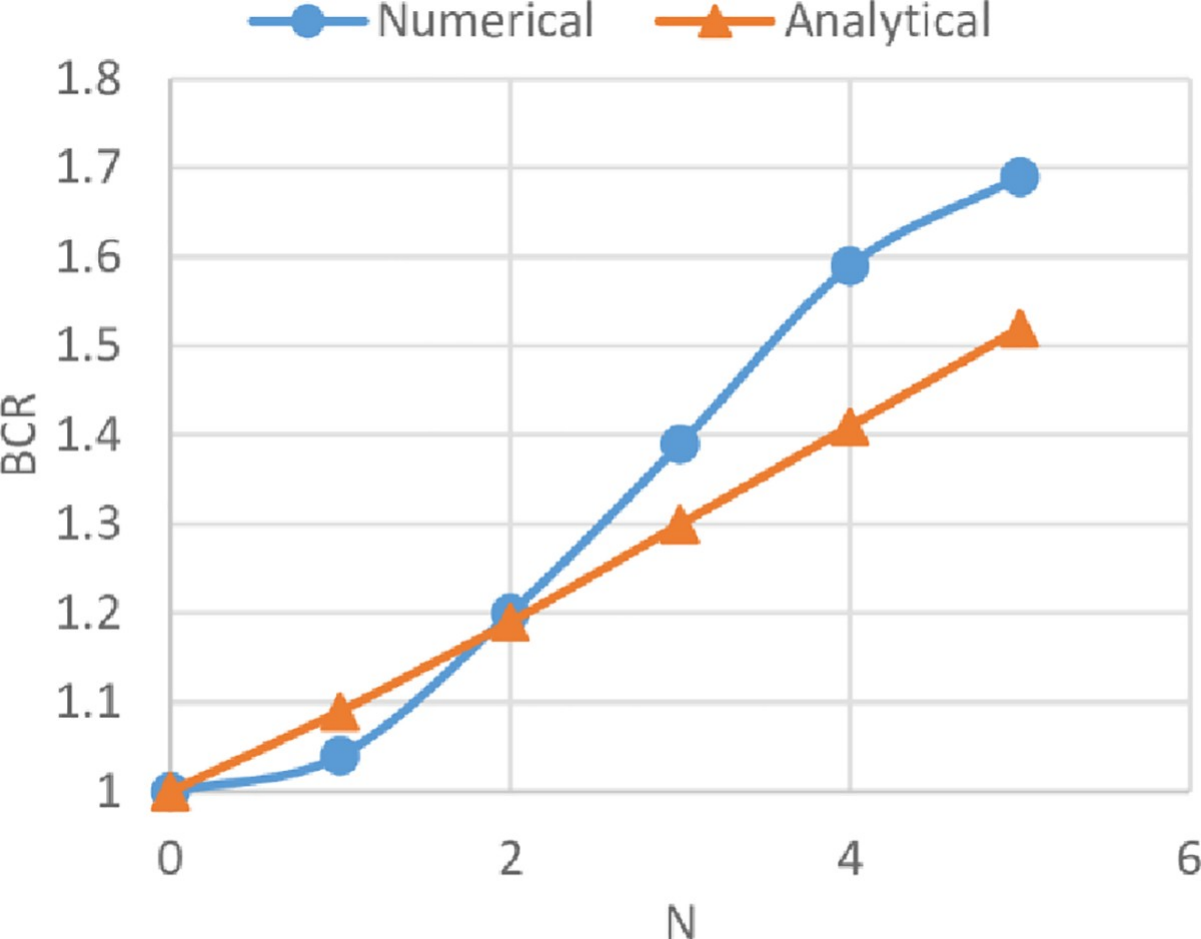
Comparison between the numerical and the analytical analysis for Al-Hamedat soil.

**Fig 34:**
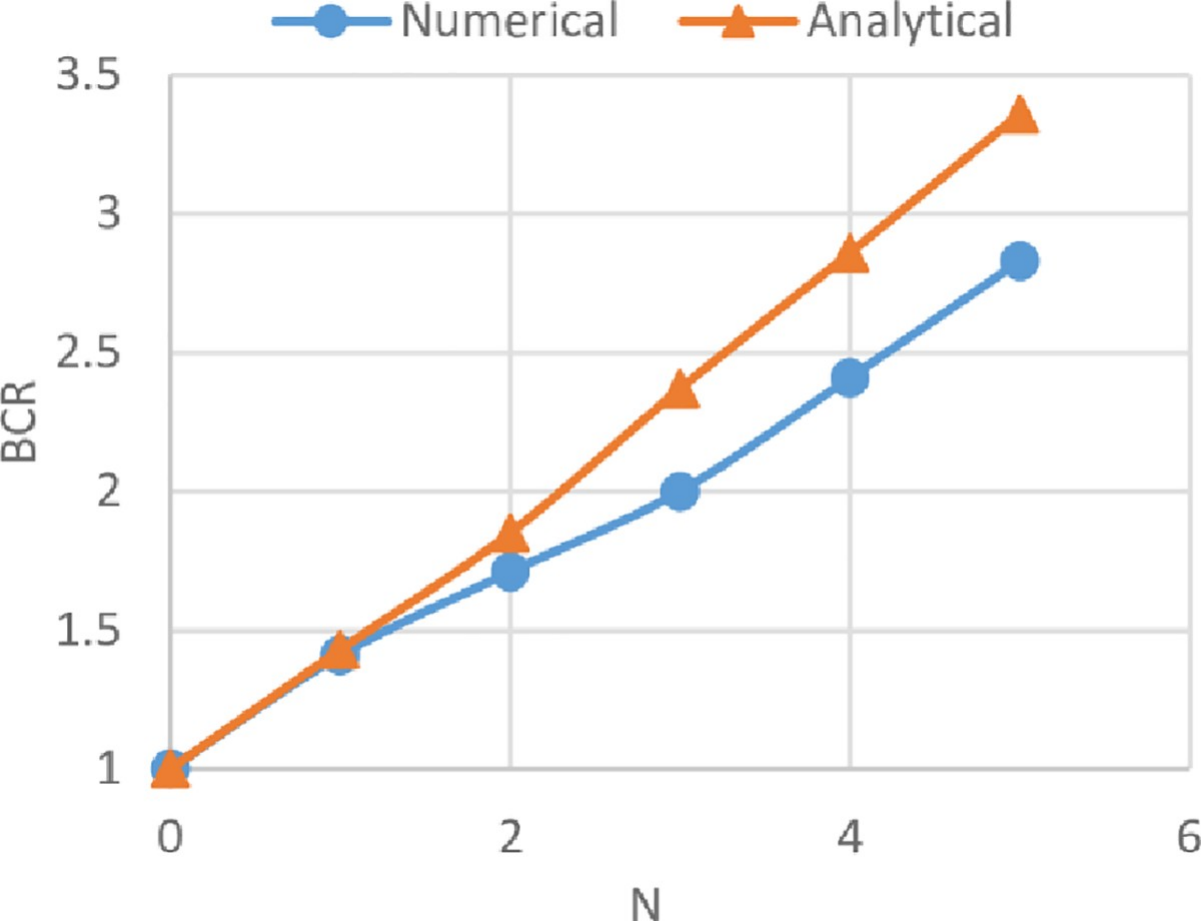
Comparison between the numerical and the analytical analysis for Al-Rashidia soil.

**Fig 35:**
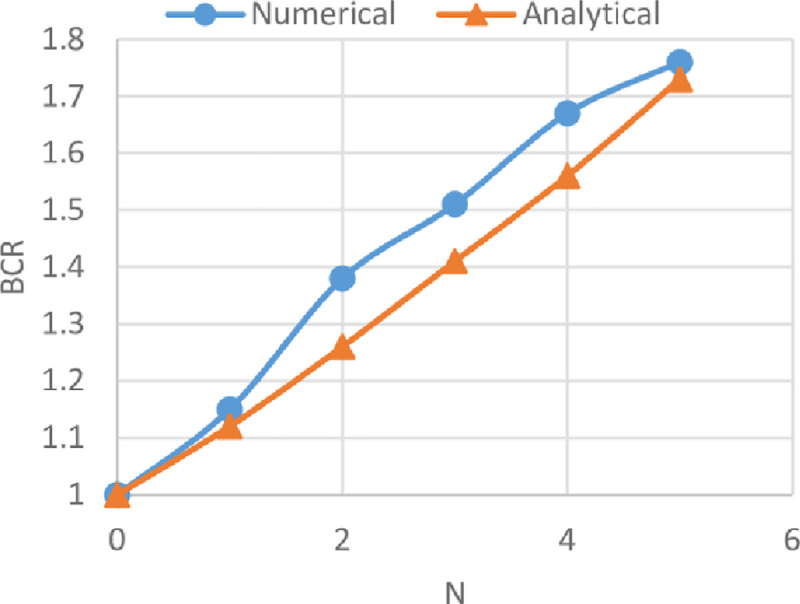
Comparison between the numerical and the analytical analysis for Ba’shiqa soil.

The BCRs from the numerical analysis by using Plaxis and from the analytical analysis by adopting the method derived by Chen and Abu-Farsakh [17] of reinforced soils of the three sites are compared in Figs 33–35. These figures show the variation of BCR of the numerical and the analytical analysis with the geogrid number (*N*) for Al-Hamedat, Al-Rashidia, and Ba’shiqa soils, respectively.

From Figs 33 to 35, it is noticeable that the analytical analysis is almost linear and showed a slight difference with a numerical analysis which might be due to the limitations in determining the exact depth of the punching shear within the clayey soils (Al-Hamedat & Ba’shiqa sites), subsequently results in low or high soil resistance to the applied loads. Also, the geogrid reinforcement inclination angle (ξ & α) values for clayey sites (Al-Hamedat & Ba’shiqa) and sandy site (Al-Rashidia) under the footing load might not exactly be selected as they are in real. However, the overall analytical analysis showed almost good results which they are close to the numerical analysis.

## Conclusion

In regards to the comprehensive finite element and analytical analysis, the inclusion of reinforcement can improve the footing’s bearing capacity and reduce the settlement. The bearing capacity and the reduction in settlement of the reinforced soil foundation for the three sites increased with an increase in the geogrid layers’ width (*b*). The degree of improvement in the bearing capacity and the footing settlement for each site was different. The soil of the Al-Hamedat site showed lower improvement than the other two sites, while the soil of the Al-Rashidia site showed higher improvement. The optimum geogrid width for all the three sites was (5*B*). An increase in the number of geogrid layers (*N*) led to an improvement in the bearing capacity and reduced the settlement of the reinforced soil foundation for all three sites. As the geogrid number increased, the degree of improvement in the bearing capacity and footing settlement for each site was different. The soil of the Al-Hamedat site showed lower improvement than the other two sites, while the soil of the Al-Rashidia site showed higher improvement. There was no optimum geogrid number, as the three sites showed good improvement even at *N* = 5. The use of geogrid reinforcement with sandy soils or weak clays beds led to better improvement in the bearing capacity and the settlement reduction than the stronger beds, which need higher settlement to show their improvements; this was unreliable because the shallow foundations were almost designed for a certain settlement level. The BCRs from the analytical analysis increased as the number (*N*) and the width (*b*) of geogrid increased. Their increment was almost linear and showed acceptable values, which were in close agreement to the BCRs from the numerical analysis. This study significantly proves that the geogrid reinforcement potentially induces improvement to the soil foundation, however, not directly subject to the width and number of the geogrid alone. The varying soil properties and footing size also contribute to both BCR and SRR values. The overall findings complemented to the advantage of applying reinforced soil foundations effectively.

## Supporting information

S1 Table Atterberg limits and grain size analysis of the three sites’ soils. (DOCX)
