# Supplementary material for: Effectiveness of strip footing with geogrid reinforcement for different types of soils in Mosul, Iraq
Source: PLoS One. 2020 Dec 17;15(12):e0243293. doi: 10.1371/journal.pone.0243293 (PMC7746198; doi:10.1371/journal.pone.0243293)
Supplement: S1 Table — (DOCX) [file pone.0243293.s001.docx]

S1 Table. Atterberg limits and grain size analysis of the three sites’ soils.

| Location | W/C % | $\gamma_{d} KN/m^{3}$ | PI % | Grain size analysis |
| --- | --- | --- | --- | --- |
| Al-Hamedat | 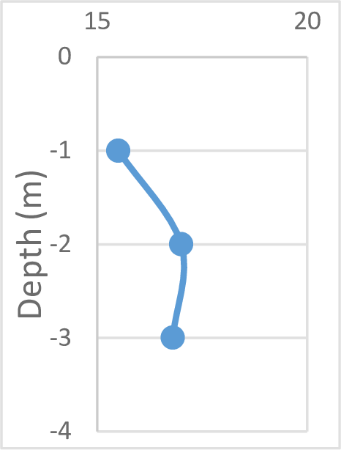 | 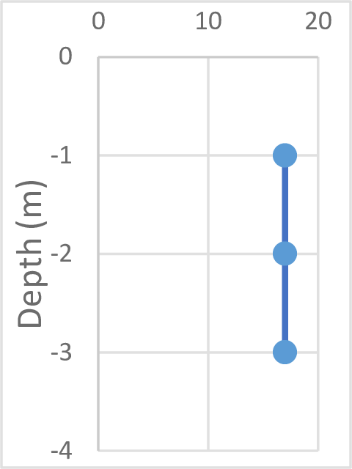 | 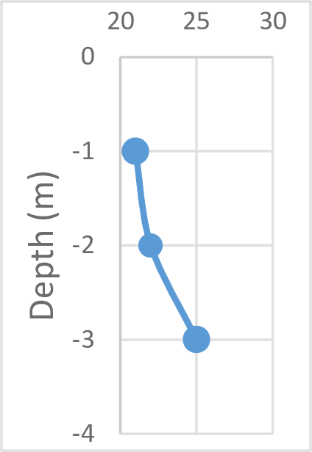 | 47% clay 45% silt at 1m depth.  47% clay 45% silt at 2m depth. |
| Ba'shiqah | 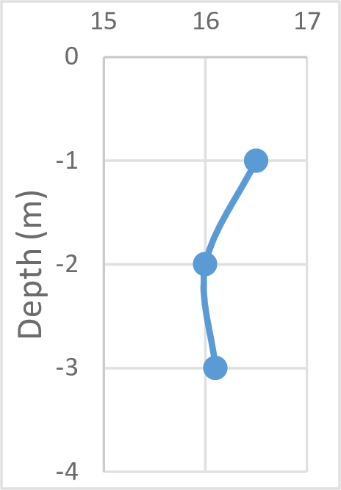 | 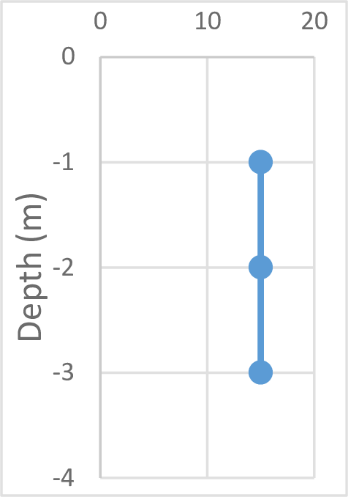 | 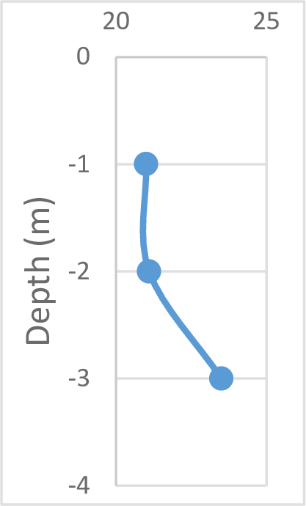 | 46% clay, 54% silt at 1m depth. 33% clay, 58% silt, 9% sand at 2m depth. 36% clay, 57% silt, 7% sand at 3m depth. |
| Al-Rashidia | 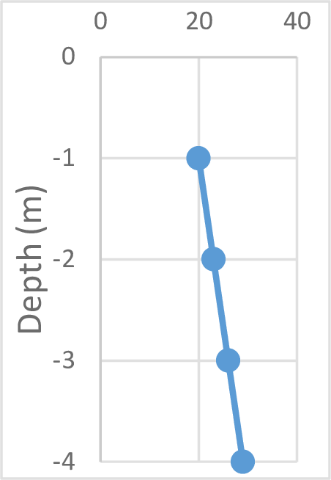 | 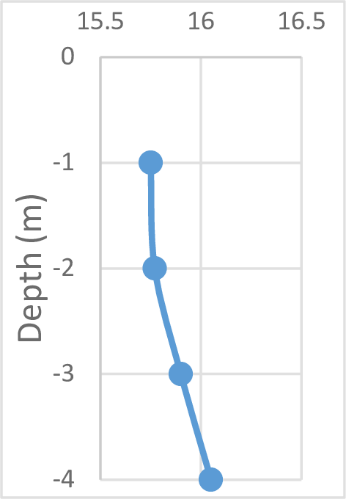 |  | 19% clay, 46% silt, 35% sand at 1m depth. 12% clay, 40% silt, 48% sand at 2m depth. 0.5% clay, 7.5% silt, 92% sand at 4m depth. |
